# Effect of Phytopreparations Based on Bioreactor-Grown Cell Biomass of *Dioscorea deltoidea*, *Tribulus terrestris* and *Panax japonicus* on Carbohydrate and Lipid Metabolism in Type 2 Diabetes Mellitus

**DOI:** 10.3390/nu13113811

**Published:** 2021-10-26

**Authors:** Maria N. Povydysh, Maria V. Titova, Igor M. Ivanov, Andrey G. Klushin, Dmitry V. Kochkin, Boris A. Galishev, Elena V. Popova, Dmitry Yu. Ivkin, Vladimir G. Luzhanin, Marina V. Krasnova, Natalia V. Demakova, Alexander M. Nosov

**Affiliations:** 1 Faculty of Pharmacy, Saint Petersburg State Chemical Pharmaceutical University, 14, Prof. Popov Str., 197376 Saint-Petersburg, Russia; dmitry.ivkin@pharminnotech.com (D.Y.I.); vladimir.luzhanin@pharminnotech.com (V.G.L.); marina.krasnova@pharminnotech.com (M.V.K.); demakova.natalya@pharminnotech.com (N.V.D.); 2 K.A. Tymyryazev Institute of Plant Physiology, Russian Academy of Sciences, 35, ul. Botanicheskaya, 127276 Moscow, Russia; struktura-500@rambler.ru (I.M.I.); andreyklyushin@list.ru (A.G.K.); dmitry-kochkin@mail.ru (D.V.K.); elena_aygol@hotmail.com (E.V.P.); al_nosov@mail.ru (A.M.N.); 3 Faculty of Biology, Lomonosov Moscow State University, 1-12 Leninskie Gory, 119234 Moscow, Russia; 4 Institute of Natural Sciences and Mathematics, Ural Federal University Named after the First President of Russia B. N. Yeltsin, 620026 Ekaterinburg, Russia; galishev@mail.ru

**Keywords:** adrenaline model, bioreactors, furostanol-type glycosides, hyperglycemia, plant cell culture, streptozotocin-induced type 2 diabetes mellitus

## Abstract

In the present study, we explored the therapeutic potential of bioreactor-grown cell cultures of the medicinal plant species *Dioscorea deltoidea*, *Tribulus terrestris* and *Panax japonicus* to treat carbohydrate metabolism disorders (CMDs) in laboratory rats. In the adrenaline model of hyperglycemia, aqueous suspensions of cell biomass pre-administered at a dose of 100 mg dry biomass/kg significantly reduced glucose level in animal blood 1–2.5 h (*D. deltoidea* and *T. terrestris*) or 1 h (*P. japonicus*) after adrenaline hydrochloride administration. In a streptozotocin-induced model of type 2 diabetes mellitus, the cell biomass of *D. deltoidea* and *T. terrestris* acted towards normalization of carbohydrate and lipid metabolism, as evidenced by a significant reduction of daily diuresis (by 39–57%), blood-glucose level (by 46–51%), blood content in urine (by 78–80%) and total cholesterol (25–36%) compared to animals without treatment. Bioactive secondary metabolites identified in the cell cultures and potentially responsible for their actions were deltoside, 25(S)-protodioscin and protodioscin in *D. deltoidea*; furostanol-type steroidal glycosides and quinic acid derivatives in *T. terrestris*; and ginsenosides and malonyl-ginsenosides in *P. japonicus.* These results evidenced for high potential of bioreactor-grown cell suspensions of these species for prevention and treatment of CMD, which requires further investigation.

## 1. Introduction

Nowadays special attention is paid to the prevention and treatment of the diseases associated with carbohydrate metabolism disorders (CMDs). These disorders impose the risk of developing diabetes and cardiovascular disease (CVD), such as hypertension, coronary heart disease, stroke, etc., which are the leading causes of deaths from the noncommunicable diseases (NCDs) [1].

The World Health Organization (WHO) named diabetes mellitus among the most impactful NCDs, and by 2030, diabetes is predicted to be the seventh cause of death worldwide. According to the WHO Global Diabetes Report, the incidence and prevalence of diabetes has been steadily increasing over the past several decades in all world regions [1,2]. In 2014, 422 million adults (8.5% of the population) had diabetes compared to 108 million (4.7%) in 1980, which implies almost four-fold increase within 35 years. Moreover, diabetes “epidemy” has serious health and socioeconomic consequences, especially in developing countries. About 40% of the annual number of deaths caused by this disease occurs in adults of working age. Particularly alarming is increasing prevalence of diabetes, especially in combination with CVD, among young age groups [1,3]. Diabetes often leads to serious complications such as heart attack, stroke, blindness, renal failure and lower limb amputation. Even at the initial stage, CMD increases the chances of life-threatening CVD complications to a high level. The risk of CVD development in men with diabetes mellitus is two to three times higher, and in women, it is 3–5 times higher, compared to persons with normal carbohydrate metabolism [4,5]. Regretfully, over 50% of the population at risk is not aware of their CMD, since it may progress for years without visible clinical symptoms.

Russia is one of the leaders among European countries in terms of mortality from diabetes mellitus [1,6,7]. Hence, early diagnosis, prevention and improving the lives of patients diagnosed with diabetes are a matter of topical interest, as declared by the national healthcare program. Adequate diabetes therapy is likely to help reduce the incidence of cardiovascular complications and increase the survival rate of this category of patients [8].

In view of the foregoing, the search for and development of new pharmaceuticals with systemic action for the prevention and correction of CMD are of significant importance. In recent years, growing attention has been paid to herbal preparations as complex and safe remedies with multifaceted cumulative action [9,10]. This interest is supported by thousands of years of herb application in traditional medicine combined with the rapid pace of modern research and development and production technologies [11,12]. The focus of modern plant-based pharmacology is the development of certified “natural” remedies with multivalent therapeutic activity and a proven safety profile which could be applied for the long-term without notable negative side effects [13].

The main problem that hinders wider application of plant-based pharmaceuticals is the depletion of natural plant resources [14]. Many medicinal plants are difficult to grow on plantations, and their over-collection in the wild may lead to disappearance of the entire species. An attractive alternative is sustainable production of plant materials with a high content of the desired biologically active metabolites via in vitro cultures of isolated cells and organs [15,16]. This “cell farming” technology enables season-independent, high-yield production of plant material in standardized batches that reduces or completely eliminates the need for harvesting wild-growing plants [17,18]. This is of particular importance for medicinal plant species that are endangered and protected in their natural habitats [19,20]. However, the biochemical composition and, hence, the biological activities of cell cultures may differ from what was reported for their source plants due to cell-culture specifics: the absence of organismic control, artificial growth conditions, lack of specific organelles that are involved in secondary metabolite biosynthetic pathways, etc. [21,22].

*Dioscorea deltoidea*, *Tribulus terrestris* and *Panax japonicus* are medicinal plants that are known to exhibit a large spectrum of pharmacological activities; however, their natural resources are dwindling [23,24,25]. Suspension cell cultures with the ability to produce triterpene and steroidal glycosides have been developed in our laboratory as a sustainable source of biomass and bioactive ingredients of these species [26,27,28,29,30], and their biochemical profiles have been investigated by using various methods [21,28,29,30,31]. Recently, Lagunin et al. [32] suggested high antihypoxic activity of phytopreparations based on *P. japonicus, D. deltoidea* and *T. terrestris* cell cultures that, in the case of *P. japonicus* and *T. terrestris,* exceeded the effect of the reference drug Mexidol. In addition, the cell biomass of *D. deltoidea* showed low toxicity in rats when administered orally even at high doses, up to 5 g/kg animal body weight [25].

However, bioreactor-produced cell cultures may differ from intact plants in chemical profiles, i.e., composition and concentrations of the produced bioactive compounds. For example, the cell culture of *D. deltoidea* contains only glycosides of furostanol type, while the spirostanol type glycosides were mostly found in the plant rhizomes [21]. These two types of glycosides have very different, sometimes opposite, biological activities [25]. Cell culture of *P. japonicus* accumulates mostly “acidic” ginsenosides (malonyl derivatives of dammarane ginsenosides and glycosides of oleanolic acid) by contrast to plant roots where neutral ginsenosides are prevailing [28]. Phenylethanoids are major compounds in the cell culture of *T. terrestris*, but only trace amounts of these chemicals are usually found in plants [31]. Therefore, the mechanisms of biological actions that have been demonstrated for plant extracts cannot be directly translated to cell cultures of the same species. Thorough investigations of the biological actions of the cell-culture-based phytopreparations and their comparison to the activities already reported for plant extracts are required. 

In this study, we explored the effectiveness of phytopreparations based on bioreactor-grown cell cultures of three medicinal plant species, namely *D. deltoidea*, *T. terrestris* and *P. japonicus,* in therapy and prevention of carbohydrate metabolism disorders in laboratory rats, using two models: adrenaline model of hyperglycemia and streptozotocin-induced model of type 2 diabetes mellitus (T2DM).

## 2. Materials and Methods

### 2.1. Plant Material

Suspension cell cultures of the following medicinal plant species were used:

- *Panax japonicus* (T. Nees) C.A. Mey., strain 62, in cell culture passport and some earlier publications referenced to as *P. japonicus* var. repens [33];
- *Dioscorea deltoidea* Wall. ex Griseb., super-producer of furostanol-type steroidal glycosides, strain DM-05–03 [26];
- *Tribulus terrestris* L., strain Tter8 [27].

All cell cultures were provided by the All-Russian collection of plant cell cultures at the Institute of Plant Physiology of Russian Academy of Sciences (Moscow, Russia) [22].

### 2.2. Bioreactor Cultivation of Cell Suspensions, Biomass Preparation and Phytochemical Analysis

Cell suspensions were cultured in laboratory (*T. terrestris*) and industrial (*P. japonicus* and *D.* *deltoidea*) bioreactors of the following types:

- Laboratory-scale bioreactors: 20 L (15 L working volume) glass bubble-type bioreactors custom-designed at the Department of cell biology and biotechnology, Institute of Plant Physiology, Moscow, Russia;
- Industrial-scale bioreactors: 630 L (550 L working volume) bubble-type bioreactors of 1T series (CUC “EBEE”, Yoshkar-Ola, Russia) with air supply through ring-type gas distributor ∅ 750 mm (Supplementary Materials Figure S1).

Conditions of bioreactor cultivation for each cell culture were described in details in [25,27,33].

During the cultivation process, culture growth and cell viability were regularly monitored, and growth parameters, such as specific growth rate, productivity and maximum biomass accumulation were calculated as previously described [27,34].

For each culture, cell biomass was harvested at the timepoint corresponding to maximum dry weight accumulation. Upon harvesting, cell biomass was placed on Nutsche Filters, and culture medium was removed under vacuum; then biomass was washed three times with distilled water. Cell biomass was dried on stainless-steel racks, at temperatures below 60 °C for 12–18 h. Aqueous suspensions were prepared by infusing dried cell biomass into purified water at a ratio of 1:20 for 7 h, at room temperature.

Phytochemical analysis of cell biomass for secondary metabolites was performed by using Nuclear Magnetic Resonance (NMR) spectroscopy, high-performance liquid chromatography with ultraviolet detection (HPLC–UV), high-performance liquid chromatography–electrospray ionization–mass spectrometry (HPLC–ESI–MS), ultrahigh-performance liquid chromatography–electrospray ionization–mass spectrometry (UPLC–ESI–MS) and spectrophotometry as previously described [25,27,28,29,30,33].

Identification of steroidal glycosides in biomass of *D. deltoidea* cell culture was performed by using gas chromatography–mass spectrometry (GC–MS), infrared spectroscopy (analysis of aglycones) [21,35], HPLC–UV [36], HPLC–ESI–MS [25,37] and NMR spectroscopy [30]. Quantitative analysis of steroidal glycosides was performed by using HPLC–ESI–MS [25].

Identification of triterpene glycosides in biomass of *P. japonicus* suspension cell culture was performed by using NMR spectroscopy [28,38], HPLC–UV [28] and UPLC–ESI–MS [29]. Quantitative analysis was performed by using HPLC–ESI–MS [39].

For *T. terrestris*, conditions of UPLC–ESI–MS analysis were different from previously published and are described below.

The analysis of an ethanolic extract of *T. terrestris* cell biomass was performed by using Waters Aquity UPLC (Waters, Santa Clara, CA, USA) equipped with hybrid quadrupole time-of-flight mass spectrometer XEVO QTOF (Waters, Santa Clara, CA, USA). After sample preparation according to [27], the sample (0.5 µL) was applied to an ACQUITY UPLC BEH Phenyl column (50 × 2.1 mm, 1.7 µm; Waters, Santa Clara, CA, USA). The column temperature was 40 °C, and the mobile phase flow rate was 0.4 ml/min. As the mobile phase, 0.1% (vol/vol) solution of formic acid (Merck) in water (Solvent A) and a 0.1% (vol/vol) solution of formic acid in acetonitrile (Solvent B) were used. Chromatographic separation was carried out in the gradient elution mode. During the analysis, the composition of the mobile phase was changed as follows (Solvent B, vol %): 0–1 min, 15%; 1–5 min, 15→30%; 5–15 min, 30→38%; 15–15.5 min, 38→45%; 15.5–23 min, 45%; and 23–23.5 min, 45→95%. The analysis was performed in the positive- and negative-ion detection mode (*m*/*z* range of 100–1200) with the following process parameters: ionization source temperature of 120 °C, desolvation temperature of 250 °C, capillary voltage of 3.0 kV, sample cone voltage of 30 V and a nitrogen (desolvation gas) supply rate of 600 L/h. Data were analyzed by using MassLynx software (Waters, Santa Clara, CA, USA). Identification of metabolites was performed based on fragmentation patterns of protonated molecular ions in the ionization source [29]. Quantitative evaluation of total content of furostanol-type steroidal glycosides in cell biomass of *T. terrestris* was performed by using spectrophotometry, as described in Reference [31].

**Figure 1:**
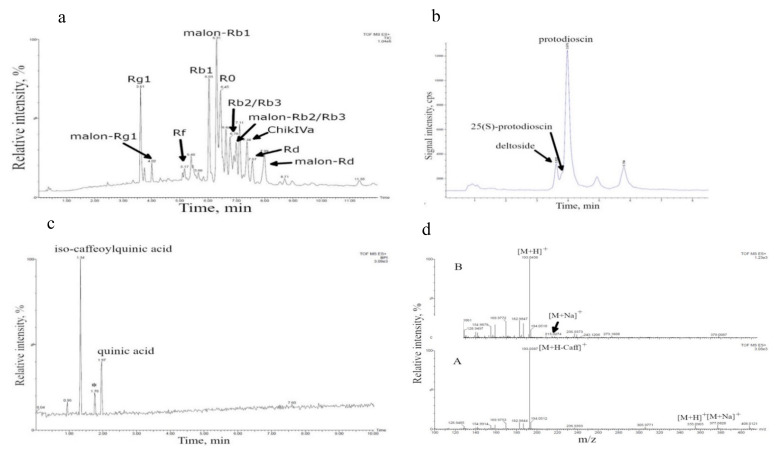
(**a**) UPLC–ESI–MS chromatogram of a methanolic extract of *Panax japonicus* suspension cell culture recorded in positive-ion mode using Waters Acquity UPLC equipped with hybrid time-of-flight mass spectrometer XEVO QTOF (Waters, USA). Chromatographic conditions are given in Reference [29]. Malon–malonyl; ChikIVa-chikusetsusaponin IVa. (**b**) HPLC–ESI–MS chromatogram of an extract of *Dioscorea deltoidea* cell suspension recorded in positive-ion mode using Agilent 1260 Infinity UPLC system equipped with a single quadrupole mass-selective detector (Agilent Technologies). Chromatographic conditions are given in Reference [25]. (**c**) UPLC–ESI–MS chromatogram of an ethanolic extract of *Tribulus terrestris* cell suspension recorded in BPI regime, positive-ion mode using Waters Acquity UPLC equipped with quadrupole time-of-flight mass spectrometer XEVO QTOF (Waters, USA). Chromatographic conditions are given in Materials and Methods. * Is likely a product of degradation of stationary phase of a cartridge for solid-phase extraction or a chromatographic column (polysiloxane). (**d**) MS spectra (positive-ion mode) of the major secondary metabolites found in ethanolic extract of *Tribulus terrestris* cell biomass. Conditions are described in Materials and Methods. (Top) Compound with a retention time of 1.34 min presumably corresponding to a caffeoylquinic acid izomer; (bottom) compound with retention time 1.97 min presumably corresponding to quinic acid. Caff—caffeic acid residue.

Representative UPLC–ESI–MS chromatograms of major metabolites specific for each cell culture are presented in Figure 1.

### 2.3. Evaluation of the Hypoglycemic Activity of Cell Biomass Preparations

First, the adrenaline model of hyperglycemia was used as the preliminary screening to reveal a potent hypoglycemic effect of phytopreparations and evaluate their therapeutic doses sufficient to reduce the blood-glucose concentration. This was followed by evaluating the effects of phytopreparations in the type 2 diabetes mellitus model (T2DM) where hyperglycemia was induced by a low dose of streptozotocin (STZ) against the background of a high-fat diet (HFD). This model is close in pathogenesis to human type 2 diabetes mellitus and allows for the evaluation of several key indicators of the carbohydrate and lipid metabolism during the stable pathological state. Using the T2DM model, we evaluated the effect of the phytopreparations on body weight, daily urine, blood-glucose concentration, blood content in urine, total cholesterol and serum triglycerides of laboratory rats. Metformin was used as a reference drug.

#### 2.3.1. Laboratory Animal Husbandry

To undertake a study, white male outbred rats obtained from the nursery of the Federal State Unitary Enterprise “Nursery of laboratory animals” Rappolovo weighing 200–250 g, and male Brown Norway Rat rats weighing 268–407 g of their own breeding were used. The animals were kept in standard conditions in accordance with Russian State Standard (GOST) R 53434-2009 “Principles of Good Laboratory Practice” and Order of the Ministry of Health of the Russian Federation dated 1 April 2016 No. 199n “On Approval of the Rules of Good Laboratory Practice”. The animals received food “Complete feed for laboratory animals” (LLC “Laboratorkorm”, Moscow, Russia) and water that meets the requirements of GOST 2874-82 “Drinking water”. Access to food and water was *provided ad libitum*, and food restriction was applied prior to blood collection. Before the start of the study, the animals were kept in a special room, in quarantine, for 14 days. Each animal was assigned an individual number (a mark applied to the tail area with a permanent indelible marker, periodically updated).

#### 2.3.2. Collection and Evaluation of Biological Material

The body weight of the animals was measured by using laboratory scales Sartogosm CE 323-C (LLC Sartogosm, Saint Petersburg, Russia) with an accuracy of 1 g.

Blood sampling for blood-glucose-level measurement and biochemical analysis was performed by incising the gum between the lower incisors. After fixing the rat by hand, blood samples were collected with a sharp blade by making a small incision in the gums (mixed blood). The blood was applied to glucometer test strips and/or collected in vacuum tubes with a blood-coagulation activator.

Blood-glucose concentration was measured by using Accu-Chek Active glucometer (Roche Diagnostics, Basel, Switzerland). In the model of adrenaline hyperglycemia, measurements were carried out in animals within the assigned time intervals, in the model of diabetes mellitus 2 (high-fat diet + STZ), measurements were carried out in animals after an overnight fast for 10–12 h. A blood volume of 1 to 2 μL was applied to a green square of a single use electrochemical strip placed in a glucometer guide. The measurement results were recorded with an accuracy of 0.1 mmol/L.

To measure total cholesterol (TC) and serum triglycerides (TG) levels, blood sampling was performed in animals after an overnight fast for 10–12 h. After taking blood from the gums into Vacuette vacuum tubes with a blood coagulation activator, the tubes were left for 30 min to settle, and then the blood was centrifuged for 10 min at 1000 rpm; the resulting serum was separated and then centrifuged again for 15 min at 4000 rpm. The analysis of the obtained serum was carried out by using a biochemical blood analyzer.

The collection of urine was carried out within 24 h in metabolic cages, ensuring the separation of feces and urine. The volume of daily urine was determined by using laboratory volumetric glassware. The concentration of glucose in urine was determined by using a urine analyzer (CL-50 Plus, High Technology Inc., North Attleborough, MA, USA) and diagnostic test strips UrineRS A10 (High Technology Inc., North Attleborough, MA, USA). The test strip was completely immersed in the reagent zones in a container with urine, in order to avoid strong dissolution of the reagent zones; the strip was quickly removed by passing it with its tip along the edge of the container with the sample to remove excess urine. After that, the strip was placed in the urine analyzer, with the reagent zones facing up; the measurements were carried out for 60 s.

At the end of the experiment, the following organs were harvested from euthanized animals: heart, liver, pancreas, kidneys, spleen and a site of skeletal muscle. The samples were washed with physiological saline solution and weighed.

#### 2.3.3. Adrenaline Model of Hyperglycemia

The model was run on white outbred male rats (N = 60), divided into 6 groups (*n* = 10). In rats of five groups, adrenaline hyperglycemia was induced by peritoneal administration of 0.1% adrenaline hydrochloride solution (Federal State Unitary Enterprise “Moscow Endocrine Plant”, Moscow, Russia) at a dose of 1 mg/kg. The sixth group was the control group. The rats were orally administered drugs 30 min before simulating hyperglycemia. Doses of administered aqueous suspensions of cell cultures were calculated based on dry cell biomass per kg of animal body weight and corresponded to 100 mg/kg. The first group of rats (*n* = 10) received metformin (Glucophage^®^, Merck Sante S.A., Darmstadt, Germany) orally at a dose of 300 mg/kg [40]. The second group (*n* = 10) received an aqueous suspension of *D. deltoidea* cells orally at a dose of 100 mg/kg. The third group (*n* = 10) received a suspension of *T. terrestris* cells orally at a dose of 100 mg/kg. The fourth group (*n* = 10) received a suspension of *P. japonicus* cells orally at a dose of 100 mg/kg. The fifth (*n* = 10) and sixth (*n* = 10) groups received an equivalent volume of 0.9% sodium chloride. The doses of cell samples were selected based on the results of the previous study [32].The blood-glucose level was measured before the start of the experiment and after oral administration of cell preparation or metformin every 30 min for 2.5 h.

#### 2.3.4. Experimental Type 2 Diabetes Model (T2DM)

The modeling was accomplished on male Brown Norway Rat rats (*n* = 25). The animals were fed a high-fat hypercaloric diet for 6 weeks. Feed composition: 73% standard feed, 25% refined edible coconut oil and 2% cholesterol [41]. After 6 weeks, animals (*n* = 20) were injected with 0.35% streptozotocin (STZ) solution at a dose equivalent to 35 mg STZ/kg animal weight [42]. STZ solution was prepared by dissolving STZ (Sigma-Aldrich, St. Louis, MO, USA) in fresh citrate buffer (pH = 4.5) in an opaque container. Citrate buffer was prepared according to Reference [43].

After receiving the STZ solution, the animals received 5% glucose solution instead of drinking water for 48 h in order to compensate for the excessive secretion of insulin from the destroyed β-cells of the pancreas, to avoid hypoglycemia and mortality from intoxication. The blood-glucose level was measured 24, 48 and 72 h after STZ administration; the urine-glucose level was determined after 48 and 72 h; the TC and TG levels—after 72 h.

After 72 h, the animals (*n* = 20) were divided into 4 groups (*n* = 5) by randomization. Group 1 received metformin at a dose of 300 mg/kg; groups 2 and 3 received suspensions of *D. deltoidea* and *T. terrestris* cells, respectively, at a dose of 100 mg/kg. Both metformin and cell preparation were administrated orally once a day for 3 weeks. The control (animals with pathology without treatment—group 4) and intact (healthy animals without pathology—group 5) groups received an equivalent volume of 0.9% sodium chloride solution. The high-fat diet continued throughout the treatment period. The study design is presented in Table 1. Blood-glucose concentration (mmol/L) was used as an indicator of hypoglycemic activity. Glucose measurements were performed after 1 and 2 weeks of receiving metformin or cell-preparation treatments, since it was expected that the hypoglycemic effect of the latter can be fully accomplished after repeated administration over time.

The level of diuresis was evaluated only in the STZ-induced model of diabetes, since, in the adrenaline hyperglycemia model, adrenaline may decrease diuresis in rats that cannot be translated into human practice. By contrast, in an STZ-induced model of diabetes, diuresis usually increases, reflecting the progress of the pathological state and is usually considered as a useful parameter to evaluate the effectiveness of treatments.

### 2.4. Statistical Data Processing

Statistical analysis of the data was performed by using the GraphPad Prism 8 software package (GraphPad Software, San Diego, CA, USA). When assessing the reliability of differences between the test groups, the hypothesis of the normal distribution of the trait was tested by using the Kolmogorov–Smirnov test; in the case of a normal distribution, the parametric Student’s *t*-test was used; if the distribution was different from the normal distribution, the nonparametric Mann–Whitney tests were applied.

## 3. Results

### 3.1. Cell Culture Growth in Bioreactors and Their Biochemical Composition

Growth, cell viability and biosynthetic characteristics were analyzed for cell cultures of *T. terrestris, P. japonicus* and *D. deltoidea* grown in bioreactors, under a semi-continuous regime. Growth characteristics for cell cultures are presented in Table 2. Since the cell cultures of three species were maintained in our laboratory of the long time (over 40 years for *D. deltoidea*, over 20 years for *P. japonicus* and five years for *T. terrestris*), their biochemical profiles have been investigated by using a variety of methods and reported earlier [21,28,29,30,31]. The results of these biochemical investigation of the cell cultures are summarized in Table 3.

The main compounds identified in the suspension cell culture of *P. japonicus* were triterpene glycosides (total content over 3% of the dry weight) with 20(S)-protopanaxadiol (ginsenosides Rb1, malonyl-Rb1, Rb2/Rb3, malonyl-Rb2/Rb3, Rd, malonyl-Rd), 20(S)-protopanaxatriol (ginsenosides Rg1, malonyl-Rg1, Rf) and oleanolic acid (ginsenoside R0 and chikusetsusaponin IVa) as aglycones (Table 3). Identification of these compounds was performed by comparing the chromatographic (HPLC–UV) behavior of glycosides with respective glycoside standards and based on the results of LC–MS analysis [29] as well as by isolation of individual triterpene glycosides and their structural identification by ^1^H- and ^13^C-NMR spectroscopy, including two-dimensional NMR experiments: ^1^H-^1^H COSY, TOCSY, NOESY, ^1^H-^13^C HSQC and HMBC and high-resolution mass spectrometry [28,38]. 

Figure 1a shows a representative UPLC–ESI–MS chromatogram (recorded in a positive-ion mode, total ion current (TIC) regime), of the methanolic extract of *P. japonicus* cell biomass harvested from a bioreactor. Identification of chromatographic peaks was performed based on the interpretation of MS1 (positive ions) spectra of triterpene glycosides (see Reference [29] for detailed method description and the results of MS1 interpretation). Mass spectra of chromatographic peaks of individual ginsenosides indicated in Figure 1a are presented in Supplementary Materials Table S1.

The main compounds found in *D. deltoidea* cell culture (over 4% of dry weight) were furostanol-type steroidal glycosides deltoside (protodeltonin), 25(S)-protodioscin (protoneodioscin) and protodioscin with diosgenin and yamogenin as aglycones (Table 3 and Figure 1b). Identification of these compounds was performed by using gas chromatography–mass spectrometry (GC–MS) and infrared spectroscopy of the products of complete acid hydrolysis of glycosides (aglycones) [21,35], comparison of the chromatographic behavior (HPLC–UV) of glycosides with a protodioscin standard [36], LC–ESI–MS analysis of individual glycosides [25,37] as well as by isolation of individual compounds from cell culture and their structural identification by ^1^H- and ^13^C-NMR spectroscopy, including two-dimensional NMR experiments ^1^H-^1^H COSY, TOCSY, NOESY, ^1^H-^13^C HSQC and HMBC, and high-resolution mass spectrometry [30]. Figure 1b shows HPLC–ESI–MS chromatogram (positive-ion mode, selected ion monitoring (SIM) regime) for ions with *m*/*z* 1047.5 and 1031.5 [25] in the extract of the *D. deltoidea* cell culture grown in 630 L bioreactor. Identification of deltoside (protodeltonin), protodioscin and 25(S)-protodioscin peaks was made based on *m*/*z* of ions [M + H − H_2_O]^+^ 1047.5 for protodeltonin and 1031.5 for protodioscin and 25(S)-protodioscin, respectively.

Furostanol-type steroidal glycosides were previously identified in the suspension cell culture of *T. terrestris* grown in flasks [27,31]. However, according to the results of spectrophotometry, these compounds are present in the cell culture grown in the bioreactor only in trace amounts, below 0.1% of dry weight. Therefore, the UPLC–ESI–MS was used to identify other metabolites with potent biological activity in the bioreactor-grown cell biomass. The results of the analysis are presented in Figure 1c,d. Two polar compounds with the retention times within 1–3 min were detected. The analysis of the mass spectra of Compound 1 with retention time of 1.34 min (positive ions, in-source fragmentation of [M + H]+) [44] drew a conclusion that the protonated molecule of this compound has an exact monoisotopic *m*/*z* value of 355.0965 which was further confirmed by the presence of the ion-adduct [M + Na]+ (exact monoisotopic value *m*/*z* = 377.0828). Therefore, we proposed that Compound 1 has the molecular formula C_16_H_18_O_9_ (calculated exact monoisotopic value for [M + H]+ (C_16_H_19_O_9_) − 355.1029, for [M + Na]+ (C_16_H_18_O_9_Na) − 377.0849). The mass spectrum of this compound also contained an intense fragment ion with *m*/*z* = 193.0387 which probably corresponded to the neutral loss of dehydrated caffeic acid residue (162 Da, C_9_H_6_O_3_). A comparison with published data [45,46] brought the conclusion that Compound 1 is an isomer of caffeoylquinic acid. Based on the analysis of the mass spectrum, Compound 2 with a retention time of 1.97 min was identified as quinic acid: [M + H]+ *m*/*z* 193.0428 and [M + Na]+ 215.0274 (calculated value for C7H13O6 − 193.0712; calculated value for C_7_H_12_O_6_Na − 215.0532). It is important to note that identification of these two compounds from *T. terrestris* cell culture was based solely on the results of liquid chromatography–mass spectrometry and requires further verification.

The compounds mentioned in Table 3 were considered as the main bioactive components of the cell cultures tested in this study.

### 3.2. Hypoglycemic Activity of Cell Culture Biomass in an Adrenaline Hyperglycemia Model

**Figure 2:**
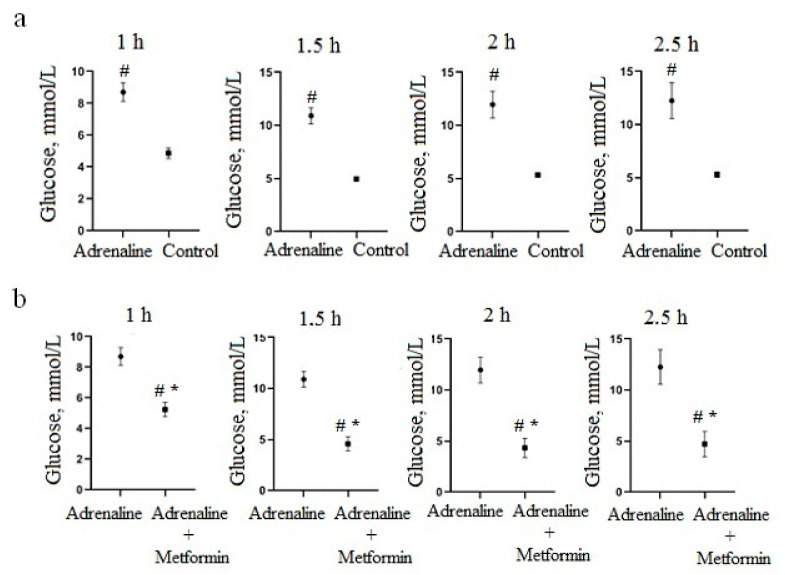
Change in glucose level in blood after administration of a peritoneal adrenaline hydrochloride solution at a dose of 1 mg/kg: (**a**) compared to a control group and (**b**) compared to group with oral pre-administration of metformin at a dose of 300 mg/kg. # Significantly different from the control group; * significantly different from the metformin group.

In an adrenaline hyperglycemia model, a significant increase in blood-glucose level was observed when adrenaline hydrochloride was administered peritoneally at a dose of 1 mg/kg compared to the control group after 1–2.5 h of administration (Figure 2a). Differences in glucose levels between the control groups and adrenaline hyperglycemia were not significant prior to adrenaline hydrochloride administration and 30 min after administration. Based on these observations, changes in glycemia level caused by phytopreparations were measured 1, 1.5, 2 and 2.5 h after administration of adrenaline hydrochloride.

Oral pre-administration of metformin at a dose of 300 mg/kg significantly reduced the capillary blood-glucose level at 1 h (*p* = 0.0005), 1.5 h (*p* < 0.0001), 2 h (*p* = 0.0003) and 2.5 h (*p* = 0.0039) after adrenaline hydrochloride administration (Figure 2b).

**Figure 3:**
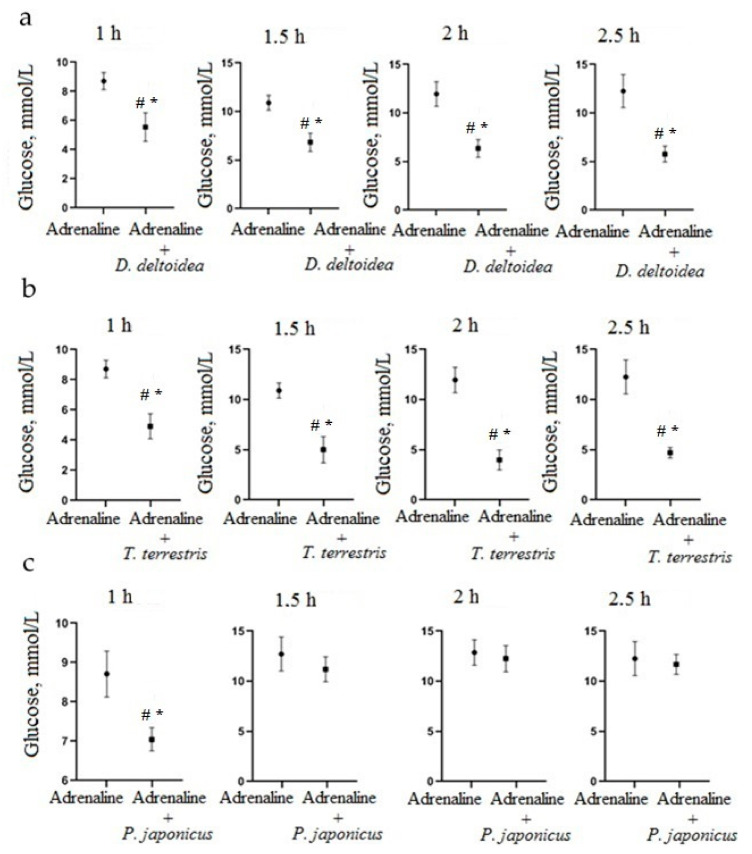
Change of glucose level in blood after a peritoneal administration of adrenaline hydrochloride solution at a dose of 1 mg/kg and oral pre-administration of suspension cells at a dose of 100 mg/kg: (**a**) *D. deltoidea*, (**b**) *T. terrestris* and (**c**) *P. japonicus*. * Significantly different from the adrenaline hydrochloride group; # significantly different from the control group.

Oral pre-administration of the aqueous suspension of *D. deltoidea* cells at a dose of 100 mg/kg significantly reduced capillary blood-glucose level at 1 h (*p* = 0.0135), 1.5 h (*p* = 0.0043), 2 h (*p* = 0.0040) and 2.5 h (*p* = 0.0059) after adrenaline hydrochloride administration (Figure 3a). Similarly, oral pre-administration of the aqueous suspension of *T. terrestris* cells at a dose of 100 mg/kg 30 min before a peritoneal adrenaline hydrochloride solution injection significantly reduced glucose level in blood at 1 h (*p* = 0.0025), 1.5 h (*p* = 0.0029), 2 h (*p* = 0.0022) and 2.5 h (*p* = 0.0274) after adrenaline hydrochloride administration (Figure 3b). Oral pre-administration of *P. japonicus* cell suspension significantly reduced the glucose level in blood only at 1 h after adrenaline hydrochloride administration (*p* = 0.0272) (Figure 3c), and then glucose concentration returned to high level. For this reason, *P. japonicus*–based phytopreparation was not included in the further stages of the study.

### 3.3. Activity of Cell Biomass Phytopreparations in Experimental STZ-Induced Model of Type 2 Diabetes Mellitus

**Figure 4:**
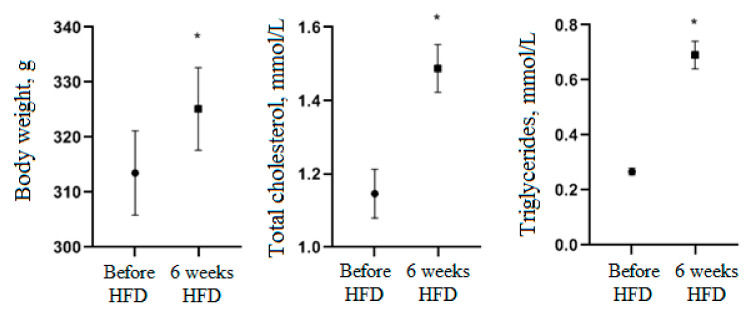
Changes in body weight of animals, total cholesterol and triglycerides in blood serum during the HFD period. * Significantly different from the period before HFD.

As a result of a six-week period of high-fat diet (HFD), body weight of animals, total cholesterol and triglycerides in blood serum significantly increased (*p* < 0.05) (Figure 4). These results suggest that HFD is effective for the development of obesity in rats, which contributes to the formation of a state of potential diabetes.

**Figure 5:**
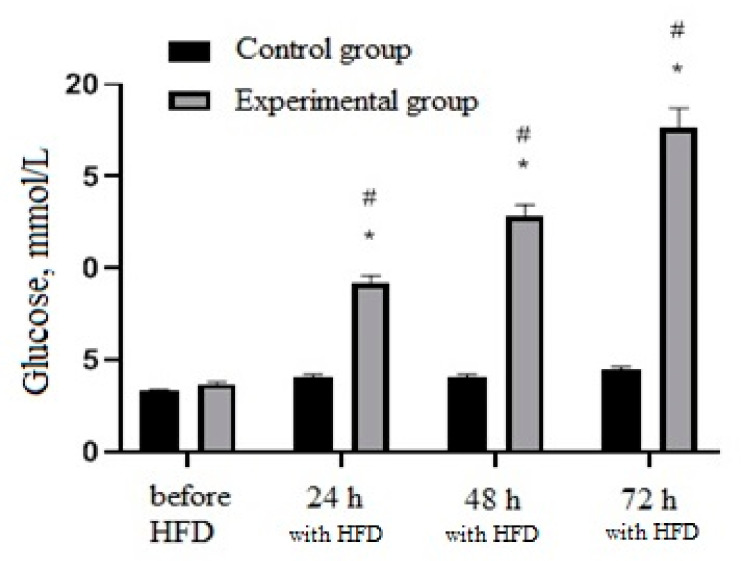
Animals blood-glucose levels measured 24, 48 and 72 h after administration of STZ (35 mg/kg). * Significantly different from the period prior to administration of STZ; # significantly different from the control group.

**Figure 6:**
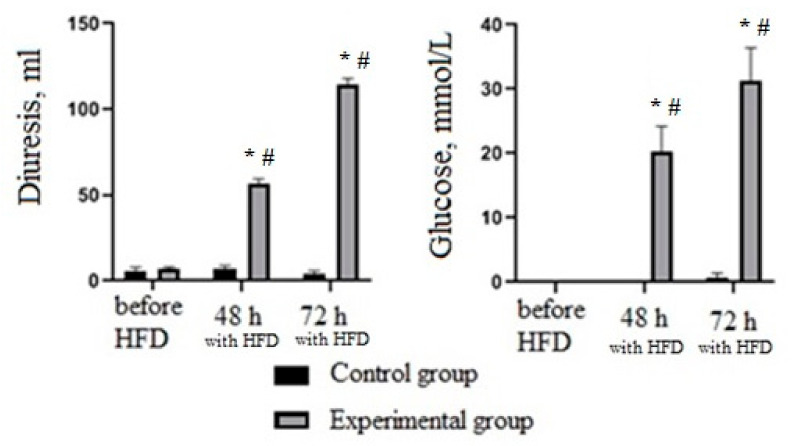
Changes in diuresis and urine-glucose level at 48 and 72 h after administration of STZ (35 mg/kg). * Significantly different from the period prior to administration of STZ; # significantly different from the control group.

In the next step, experimental type 2 diabetes mellitus was stimulated in rats by introducing STZ solution. In the experimental group blood-glucose level significantly increased (*p* < 0.05) in proportion to the time after administration of STZ. Moreover, the blood-glucose level of the experimental group significantly exceeded the values of the control group (*p* < 0.05) (Figure 5). In addition, the administration of STZ significantly increased daily diuresis and urinary glucose levels of rats measured 48 and 72 h after administration of STZ compared to the period before administration and to the control group (*p* < 0.05) (Figure 6).

**Figure 7:**
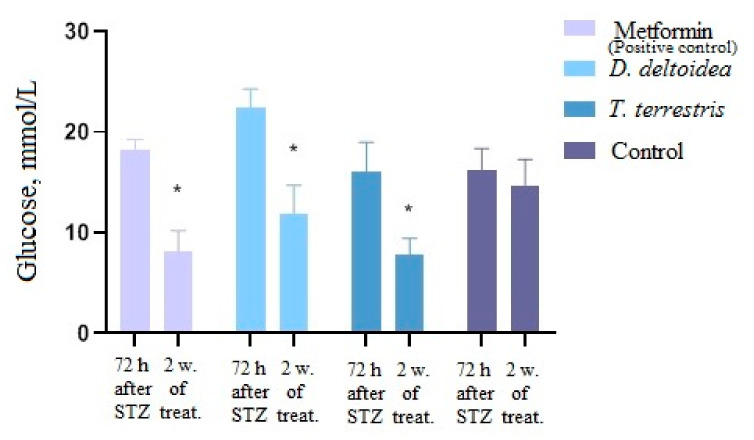
Change of glucose level in blood after treatment with metformin and phytopreparations. * Significantly different from the period after administration of STZ. Control—STZ treatment followed by administration of sodium chloride, w—weeks and treat.—treatment.

These results indicate that a single peritoneal administration of STZ at a dose of 35 mg/kg on the background of the HFD led to the formation of type 2 diabetes mellitus in rats which progressed over time and was aggravated by the HFD. Subsequent treatment of animals with *D. deltoidea–* and *T. terrestris*–based phytopreparations and metformin led to a significant (*p* < 0.05) decrease in blood-glucose level. After oral administration of metformin (300 mg/kg), the blood-glucose level decreased by 55% relative to the period after administration of STZ. Phytopreparations of *D. deltoidea* and *T. terrestris* cells also demonstrated a hypoglycemic effect by reducing glucose level in blood by 46% and 51%, respectively, compared to treatment with STZ alone (Figure 7). The effects of phytopreparations were similar (*T. terrestris*) or comparable (*D. deltoidea*) to the effect of metformin. Administration of an equivalent amount of sodium chloride did not lead to a significant decrease in glucose levels (Figure 7).

**Figure 8:**
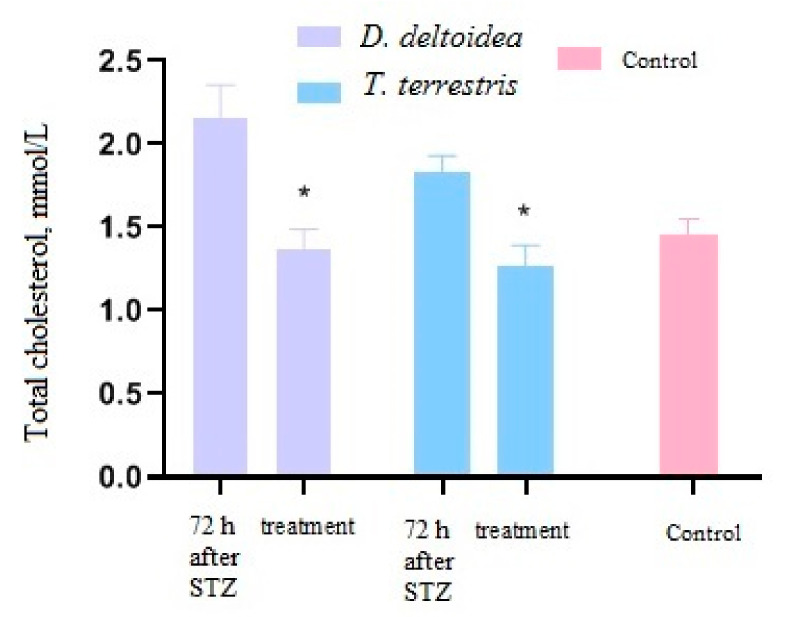
Change of total cholesterol level in blood after treatment with phytopreparations. * Significantly different from the period after administration of STZ. Control—intact animals.

Significant changes in the level of total cholesterol were only observed following administration of *D. deltoidea* and *T. terrestris* cell biomass (Figure 8) but not after metformin or NaCl solution (not showed in the figure). No reliable changes in the triacylglycerols level were observed in any of the treatments.

No significant change in body weight was recorded during the treatment period, and this may be associated with short-term treatment.

**Figure 9:**
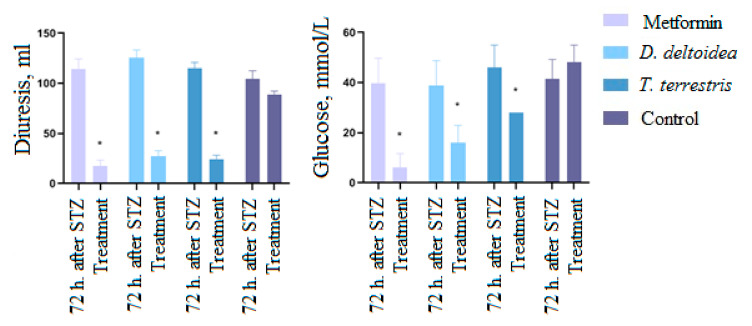
Change of diuresis and glucose level in urea after treatment with metformin and phytopreparations. * Significantly to the period after administration of STZ.

During the period of treatment with metformin and phytopreparations, diuresis and glucosuria (*p* < 0.05) significantly decreased (Figure 9). Thus, the daily urine volume of rats treated with metformin decreased by 85% relative to the period after administration of STZ; in animals treated with *D. deltoidea*–based phytopreparation, diuresis decreased by 57%, and those treated with *T. terrestris*–based phytopreparation decreased by 39%. In addition, urine-glucose concentration decreased in the group receiving metformin by 84%; in the group treated with *D. deltoidea* phytopreparation, it decreased by 78%; and in the group treated with *T. terrestris* phytopreparation, it decreased by 80%. Normalization of urinary glucose was achieved with a longer treatment period.

Therefore, the results of the study evidenced that oral administration of phytopreparations based on *D. deltoidea* and *T. terrestris* cell biomass act towards the normalization of carbohydrate and lipid metabolism in animals with type 2 diabetes mellitus.

### 3.4. Results of Histological Analysis

Histological analysis was performed on intact and treated animals in the STZ-induced model of type 2 diabetes mellitus. The following organs were examined: heart, spleen, liver, pancreas, kidneys and skeletal muscles.

Animals in the intact group and in the group treated with metformin showed no pathology during the examination. All animals of these groups had normal cardiovascular system, liver, kidneys and skeletal muscles. In two animals of the intact group, weakly expressed granulomatous hepatitis and degeneration of hepatocytes were revealed (Supplementary Materials Figure S2a,b). Similarly, no significant pathology was detected in four of five animals in the groups treated with the *D. deltoidea*– and *T. terrestris*–based phytopreparations. Seldom appearance of large lipid droplets with hepatocytes degeneration were observed in the liver of one animal from the *D. deltoidea* group (Supplementary Materials Figure S3a) and one animal from *T. terrestris* group (Supplementary Materials Figure S3b).

By contrast, within the group that received an equivalent amount of sodium chloride, pathological changes were observed in four out of five animals. These included degeneration of hepatocytes, autolysis of kidney, spleen and pancreas tissues (Supplementary Materials Figure S4).

## 4. Discussion

Phytopreparations containing plant saponins (triterpene or steroidal glycosides) have a great potential to meet the growing demand for new remedies for the treatment of diabetes mellitus type 2 due to their complex and effective action, as well as low frequency of side effects compared to many existing hypoglycemic drugs [47]. The increase in the incidence of diabetes mellitus, mainly type 2, and dangerous chronic complications caused by hyperglycemia (diabetic nephropathy, diabetic retinopathy, diabetic foot, diabetic neuropathy, atherosclerosis, etc.) necessitates extensive preclinical studies of phytopreparations to reveal their action pathways, potential side effects and effective therapeutic doses. This requires experimental models on animals most sensitive to the development of diabetes, adequate methods for inducing diabetes and the careful choice of evaluation criteria.

The literature analysis has shown that rodents (mainly mice, including wild breeds) and minipigs are best suited for modeling T2DM. The closest in etiology and development mechanisms to T2DM in humans are diet-induced models, among which high-carbohydrate diets additionally enriched with sucrose or fructose are the most effective [42,48,49]. Low doses of streptozotocin were proven effective in acceleration of the development of diet-induced type 2 diabetes due to its moderately destructive action towards β-cells of the pancreas compared to their severe damage in the case of type 1 diabetes [50]. The main criteria for the development of diabetes and the effectiveness of the investigated therapeutic and prophylactic measures currently used in the studies are glucose content, insulin and glycosylated hemoglobin of blood plasma; the number of β-cells in the islets of the pancreas; the area of distribution of the sugar curve; the index of insulin resistance; and the results of histology and histochemistry of the pancreas and other organs [51,52,53,54].

Plant saponins have been extensively studied for their antidiabetic effects [47]. Plants of *Tribulus terrestris* that contain saponins are known to have antidiabetic action [23,55,56]. The extract of *T. terrestris* tested in streptozotocin-induced diabetic rats significantly decreased the levels of alanine aminotransferase and creatinine in the blood serum and lowered the malondialdehyde level in the liver of the diabetic animals. Histopathological examination revealed significant recovery of liver in herb-treated rats [23]. Antidiabetic activity, including inhibitory effects on α-amylase and α-glucosidase, has been reported for hexane, alcohol and acetone extracts of *T. terrestris* fruits [57]. In the present study, the cell culture of this species also demonstrated antidiabetic effect in both models tested. This included the significant reduction of daily diuresis, blood-glucose level, blood content in urine and total cholesterol compared to rats with induced diabetes that did not receive phytopreparations. *T. terrestris* extracts significantly decreased the levels of ALT and creatinine in the serum of diabetic rats and lowered the MDA level in liver in both diabetic and control animal groups [23]. Histological observations revealed significant recovery of liver in rats that received the extract [23]. It is interesting, however, that the content of steroidal glycosides in *T. terrestris* cell culture was below 0.1% dry weight (Table 3). In addition, two other compounds with potent biological activity (an isomer of caffeoylquinic acid and quinic acid) were identified in the cell culture. These compounds have a wide range of biological activities, such as antioxidation, antibacterial, antiparasitic, neuroprotective, anti-inflammatory, anticancer, antiviral and antidiabetic effects [58,59]. These results support our previous hypothesis that cell cultures may produce different spectrum of metabolites compared to intact plants, and their biological activities may be attributed to mechanisms different from that reported for plants of the same species.

*Panax* species are well-studied for their antidiabetic properties [60]. Recent studies are mostly focused in revealing and evaluation of the antidiabetic activities of individual ginsenosides [9,61]. Recently, Bai et al. [62] reviewed the therapeutic effects of ginsenosides and their potential use in diabetes treatment and listed 18 effects associated with ginsenoside action. These included, among others, the improvement of insulin resistance, insulinotropic action, hepatoprotective activity, anti-inflammatory activity, myocardial protection, neuroprotection, lipid regulation, improvement of glucose tolerance, etc. The authors concluded that ginsenosides Rg1, Rg3, Rb1 and compound K are the most promising as antidiabetic compounds. Similarly, Zhou et al. [61] presented Rb1 as one of the most perspective antidiabetic substance, as it has the ability to regulate mitochondrial energy metabolism, improve insulin resistance and mitigate the occurrence of complications. *P. japonicus* saponins were reported to stimulate the production of GLP-1 and the expression of PPAR-γ, which are links in the pathogenesis of T2DM [61]. At least two compounds with potent antidiabetic activity, namely Rg1 and Rb1, among other ginsenosides and their malonylated derivatives, were identified in the cell culture of *P. japonicus* in our study. Administration of cell biomass of *P. japonicus* reduced glucose level in blood in the adrenaline hyperglycemia model but this effect was transient. These results further confirm the importance of the *in vivo* investigation of biological actions of cell cultures that can be different from those reported for plants or their individual compounds. To the best of our knowledge, the antidiabetic effects of *Dioscorea deltoidea* have never been studied in the published literature, while other *Dioscorea* species are known for their antidiabetic action [63,64]. In particular, antidiabetic effects of *D. polygonoides* plant extracts were demonstrated in streptozotocin-induced diabetic rat model [65]. The main active substance of the *Dioscorea* genus, diosgenin, possesses therapeutic properties and is used in the treatments of type 2 diabetes [10,66,67]. Its effects include, but are not limited to, hypoglycemic, hypolipidemic, anti-inflammatory, antioxidant and neuroprotective actions [10]. According to Fuller et al. [68], diosgenin exhibited the properties of a secretagogue and a sensitizer that lowered blood glucose in rodents. In the present study, the cell culture of *D. deltoidea* containing furostanol-type glycosides with diosgenin as aglycone demonstrated antidiabetic activities, which included reducing the glucose level in blood and urine, lowing the level of total cholesterol and decreasing diuresis.

The use of bioreactor-grown cell cultures of medicinal plants in preparation of phyto-remedies is a valuable alternative to over-collecting wild plants in nature [20]. The cell-based biotechnology is sustainable, environmentally friendly and season-independent, with potential for standardization of the final product [11,18,19]. However, the results of our study further confirmed that cell biomass is not a full equivalent of the whole plant and, as such, requires thorough investigation and clinical trials.

In the present work, the ability of phytopreparations based on cell cultures of *D. deltoidea, T. terrestris* and, to a lesser extent, *P. japonicus*, were shown to reduce elevated blood-glucose levels in comparison with the standard treatment for diabetes mellitus (metformin) in adrenaline hyperglycemia model in rats.

Using another model, STZ-induced T2DM, in combination with a high-fat diet, we were able to confirm the role of obesity in the formation of type 2 diabetes (indicated by an increase in the body weight of animals and an increase in the level of total cholesterol and triglycerides in blood serum). In addition, we confirmed the previously reported hypoglycemic properties of *D. deltoidea* and *T. terrestris* saponins. The same model demonstrated that herbal preparations of *D. deltoidea* and *T. terrestris* reduced daily urine output and glucosuria in animals. Moreover, phytopreparations of *D. deltoidea* and *T. terrestris* cells were proven to affect lipid metabolism in diabetes mellitus 2, in particular, to reduce the level of total cholesterol in animals continuing to receive a high-fat diet.

The pleiotropic effect of phytopreparations observed in this study is very interesting. Similarly, Qin et al. [69] noted the ability of ginsenosides to exert a positive effect on the body promoting cardiac function during DM. The potential antihypoxic effect of saponins from *D. deltoidea* and *T. terrestris* cell cultures was also reported [32].

Thus, in the present study, the prospects for further research of phytopreparations based on *D. deltoidea, T. terrestris* and *P. japonicus* were proved, due to their positive effect on a number of indicators in DM type 2 and their pleiotropic antihypoxic effect. Due to the complex biochemistry, the plant-cell-based remedies may act through multiple targets and multiple pathways, implying their potential application not only in therapy, but also as dietary supplements to help control the development of diabetes mellitus.

## 5. Conclusions

In this study, suspension cell cultures of *D. deltoidea*, *T. terrestris* and *P. japonicus* grown in bioreactors were investigated for their potential efficiency in the prevention and treatment of type 2 diabetes mellitus. Cell cultures demonstrated high growth and biosynthetic characteristics. The secondary metabolites detected in the cell cultures were deltoside, 25(S)-protodioscin and protodioscin in *D. deltoidea*; furostanol-type steroidal glycosides and quinic acid derivatives in *T. terrestris*; and ginsenosides and malonyl-ginsenosides in *P. japonicus,* thus confirming the results of earlier reported phytochemical analysis.

Preclinical models of type 2 diabetes mellitus, namely adrenaline hyperglycemia and a combination of a high-fat diet with STZ-induced T2DM, were effectively used to investigate the potential effects of phytopreparations. In the adrenaline hyperglycemia model, phytopreparations based on cell cultures of *D. deltoidea, T. terrestris* and, to a lesser extent, *P. japonicus* showed a hypoglycemic effect. In T2DM, phytopreparations based on *D. deltoidea* and *T. terrestris* reduced the level of glucose in blood and urine, daily urine volume and total cholesterol. These effects of phytopreparations were comparable to the effect of the reference drug, metformin. However, the administration of phytopreparations had no effect on body weight.

These results evidenced the high potential of bioreactor-grown cell suspensions of the selected species for the prevention and treatment of carbohydrate and lipid metabolism disorders and open the door for further studies in the future to investigate the use of these cultures in the diet of patients with high risks of developing diabetes and its complications.

## Figures and Tables

**Table 1:** Experimental design to investigate hypoglycemic activity of cell biomass of *D. deltoidea* and *T. terrestris*, using Type 2 diabetes model (T2DM).

Manipulation	Day of the Experiment ^1^							
	0 ^2^	1–42	43	44 (24 h STZ)	45 (48 h STZ)	46 (72 h STZ)	47–53 1st Week of Treatment	54–60 2nd Week of Treatment
High-fat diet		+	+	+	+	+	+	+
STZ administration 35 mg/kg			+					
Visual check for pathology development		+	+	+	+	+		
Drug administration							+	+
Body weight measurement	+	+ (21,42)				+ (60)		+ (60)
Blood-glucose-concentration measurement		+ (42)		+	+	+	+ (53)	+ (60)
Measuring the concentration of glucose in urine	+	+ (42)			+	+	+ (53)	+ (60)
Daily urine volume measurement	+	+ (42)			+	+	+ (53)	+ (60)
Blood cholesterol and triglyceride level measurement		+ (42)				+		+ (60)
Euthanasia								+ (60)
Histological examination								+ (60)

^1^ Numbers in parenthesis indicate the exact days when measurements were performed during the given time period. ^2^ Evaluations made before the beginning of the experiment. STZ—streptozotocin.

**Table 2:** Growth and viability characteristics of the suspension cell cultures of *D. deltoidea*, *T. terrestris* and *P. japonicus* grown in bioreactors. All parameters were calculated based on dry weight.

Cell Culture	Bioreactor Volume	Cell Viability, V (%)	Maximum Accumulation of Dry Weight, Mmax_dw (g/L)	Productivity, Pi_Max (g/(l⋅Day)	Specific Growth Rate, µdw, (Day^−1^)
*D. deltoidea*	630 L	83.5 ± 4.8	9.10 ± 2.76	0.37 ± 0.07	0.11 ± 0.03
*T. terrestris*	20 L	99.0 ± 1.0	14.0 ± 0.10	1.10 ± 0.20	0.3 ± 0.05
*P. japonicus*	630 L	84.2 ± 3.6	8.16 ± 1.84	0.31 ± 0.04	0.09 ± 0.02

**Table 3:** Secondary metabolites detected in the suspension cell cultures of *D. deltoidea*, *T. terrestris* and *P. japonicus* grown in bioreactors.

Cell Culture	Bioreactor Volume	Bioactive Metabolites ^1^	Total Content of Detected Bioactive Metabolites (% of Dry Cell Weight)
*D. deltoidea*	630 L	Furostanol-type glycosides (deltoside, 25 (S)-protodioscin, protodioscin) [21,30] ^2^	4.62 ± 0.53
*T. terrestris*	20 L	Furostanol-type steroidal glycosides [27,31] Caffeoylquinic acid, quinic acid (this study)	0.10 ± 0.03 not determined
*P. japonicus*	630 L	Ginsenosides Rg1, malonyl-Rg1, Rb1, malonyl-Rb1, Rb2/Rb3, malonyl-Rb2/Rb3, Rd, malonyl-Rd, Rf, R0, chikusetsusaponin IVa) [28,29]	3.46 ± 0.68

^1^ Determined at the time of harvesting corresponding to maximum accumulation of dry weight for each cell culture. ^2^ References to original studies where the presence of these compounds was first reported for each culture.

## Data Availability

No new datasets were generated during this study. Data sharing is not applicable to this article.

## Supplementary Materials

The following are available online at https://www.mdpi.com/article/10.3390/nu13113811/s1, Table S1: UPLC–ESI–MS data for the extract of *Panax japonicus* cell culture grown in bioreactors (recorded in positive-ion mode). Figure S1: 630 L bioreactors for cultivation of cell suspensions. Figure S2: Two observations of weakly expressed pathology in the liver of the intact animals: (a) minor granulomatous hepatitis and (b) scattered hepatocyte degeneration. Hematoxylin and eosin staining. Magnitude ×400. Figure S3: Occasionally observed large lipid droplets with hepatocyte degeneration in the liver of rats in the STZ-induced model of type 2 diabetes mellitus that received treatments with phytopreparations of (a) *D. deltoidea* and (b) *T. terrestris*. Hematoxylin and eosin staining. Magnification ×400. Figure S4: Various pathologies observed during histological examination in animals of the control group in the STZ-induced model of type 2 diabetes mellitus that received sodium chloride (diabetes without treatment): (a,b) medium and small lipid droplets with hepatocyte degeneration; (c) large and medium lipid droplets with hepatocyte regeneration; (d) total autolysis of kidney tissue; (e) partial autolysis of the pancreas, kidneys, spleen and lung; and (f,g) hepatocyte degeneration. Hematoxylin and eosin staining. Magnitude ×400.
