# Supplementary material for: Effect of Phytopreparations Based on Bioreactor-Grown Cell Biomass of Dioscorea deltoidea, Tribulus terrestris and Panax japonicus on Carbohydrate and Lipid Metabolism in Type 2 Diabetes Mellitus
Source: Nutrients. 2021 Oct 26;13(11):3811. doi: 10.3390/nu13113811 (PMC8617789; doi:10.3390/nu13113811)
Supplement: Supplementary file 1 [file nutrients-13-03811-s001.zip › nutrients-1313891-supplementary.pdf]

nutrients

MDPI

Supplementary Table 1. UPLC-ESI-MS data for the extract of *Panax japonicus* cell culture grown in bioreactors (recorded in positive ion mode). The analysis was performed using Waters Acquity UPLC-ESI-MS system (Waters, USA) as described in [29].

Retention time, min	$[M+H]^+$ *m/z*	Other ions *m/z*	Triterpene glycoside
3.61	801.5	783.5; 765.5; 621.4 603.4; 585.4; 441.3 423.3; 405.3	ginsenoside Rg1
4.02	887.5	869.5; 707.4; 689.4 671.4; 621.4; 441.4 423.4; 405.3	malonyl-ginsenosde Rg1
5.17	801.5	783.5; 765.5; 747.5 621.4; 441.4; 423.4	ginsenoside Rf
6.03	1109.6	1091.6; 947.6; 929.5 785.5; 425.4	ginsenoside Rb1
6.31	1195.6	1177.6; 1015.5; 853.5; 425.4	malonyl-ginsenosde Rb1
6.45	957.5	795.4; 777.4; 633.4 439.3	ginsenoside R0
6.78	1079.6	1061.6; 899.5; 767.5 755.5; 749.5	ginsenoside Rb2/Rb3
7.00	1165.6	1147.6; 985.5; 853.5 407.4	malonyl-ginsenosde Rb2/Rb3
7.38	795.5	633.4; 615.4; 439.3	chikusetsusaponin IVa
7.57	947.5	929.5; 767.5; 749.5 605.4; 425.4	ginsenoside Rd
7.99	1033.6	853.5; 835.5; 425.4 407.4	malonyl-ginsenosde Rd

Supplementary Figure 1. Industrial scale bioreactors: 630-l (550 l working volume) bubble-type bioreactors of 1T series (CUC "EBEE", Yoshkar-Ola, Russia) with air supply through ring-type gas distributor Ø 750 mm

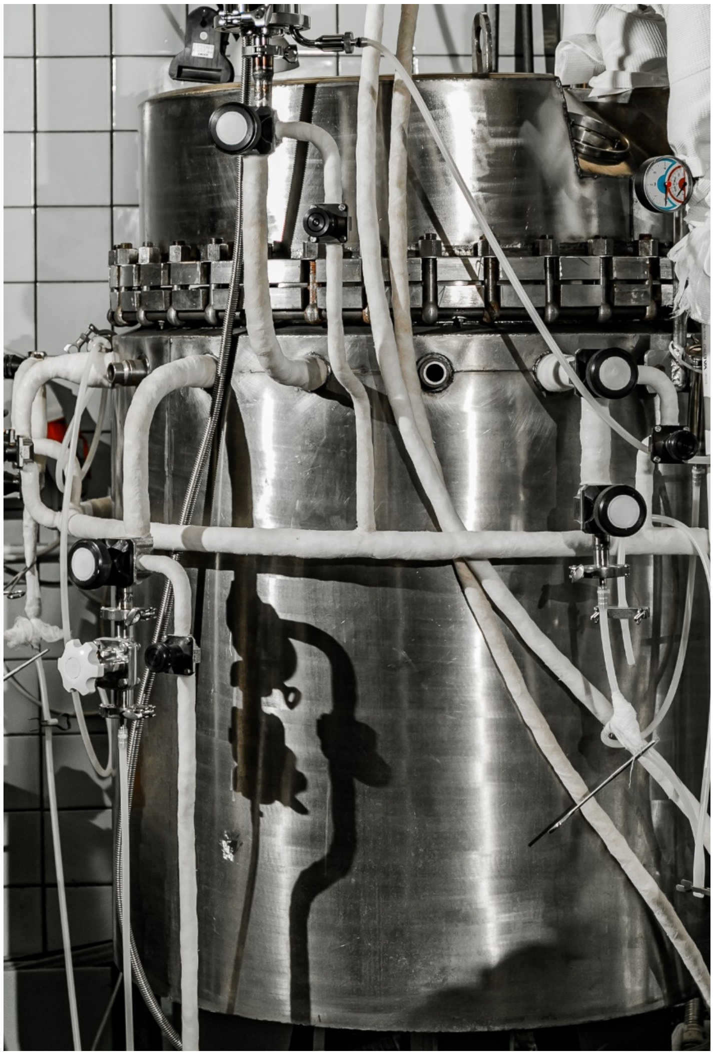

Supplementary Figure 2. Two observations of weakly expressed pathology in the liver of the intact animals. **(a)** minor granulomatous hepatitis, **(b)** scattered hepatocytes degeneration. Hematoxylin and eosin staining. Magnitude x400.

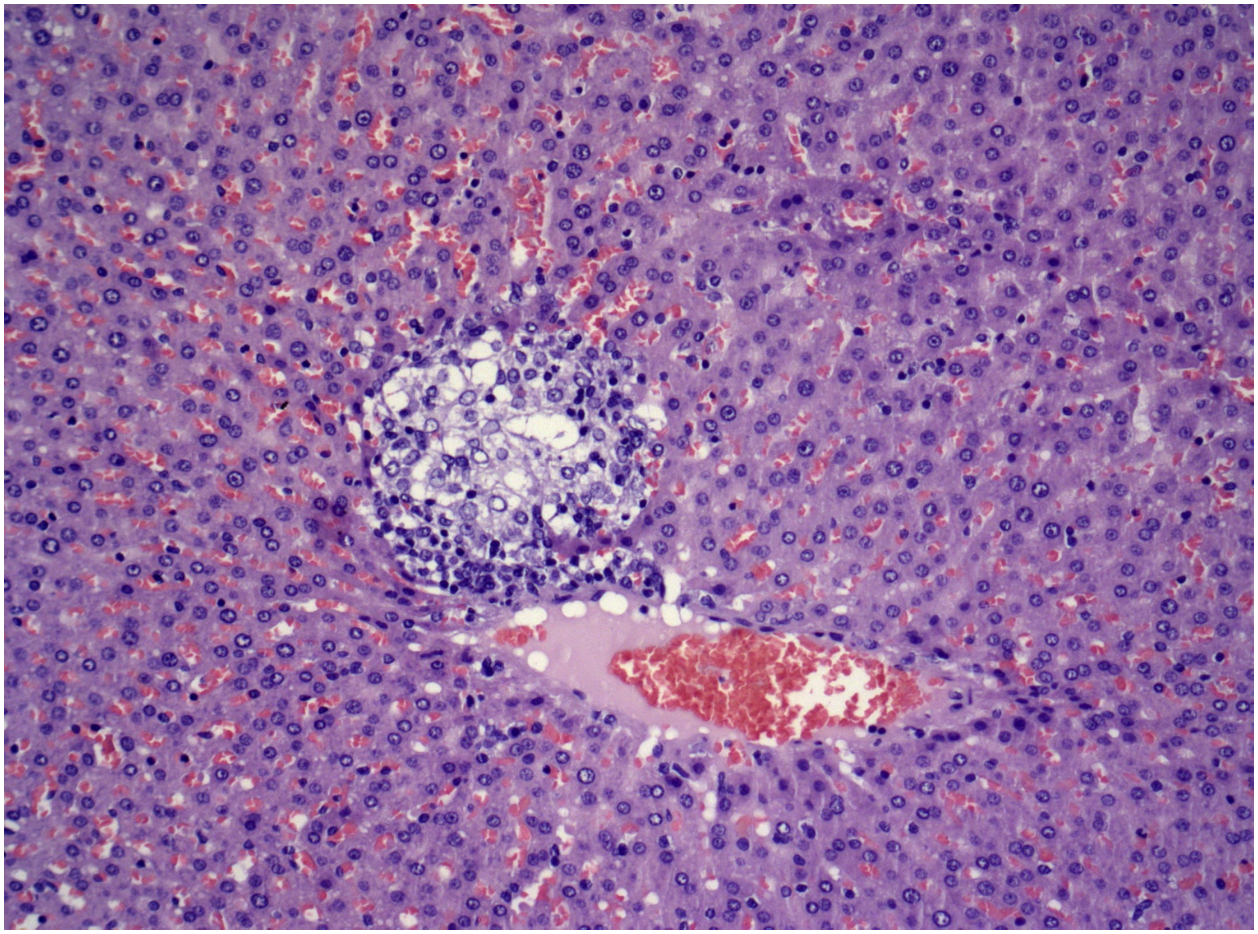

Supplementary Figure 2a

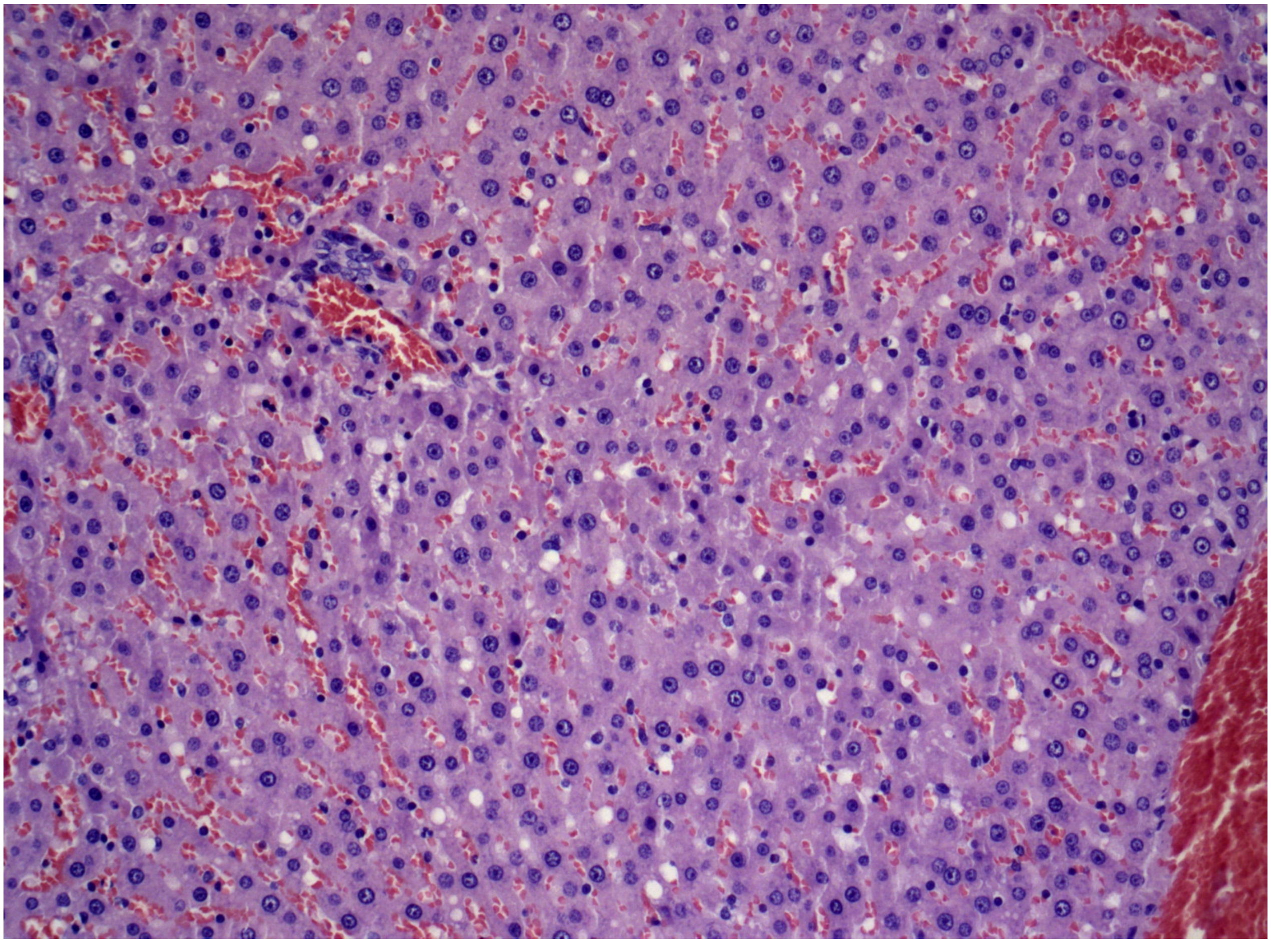

Supplementary Figure 2b

Supplementary Figure 3. Occasionally observed large lipid droplets with hepatocyte degeneration in the liver of rats in the STZ – induced model of type 2 diabetes mellitus that received treatments with phytopreparations of **(a)** *D. deltoidea* and **(b)** *T. terrestris*. Hematoxylin and eosin staining. Magnification x400.

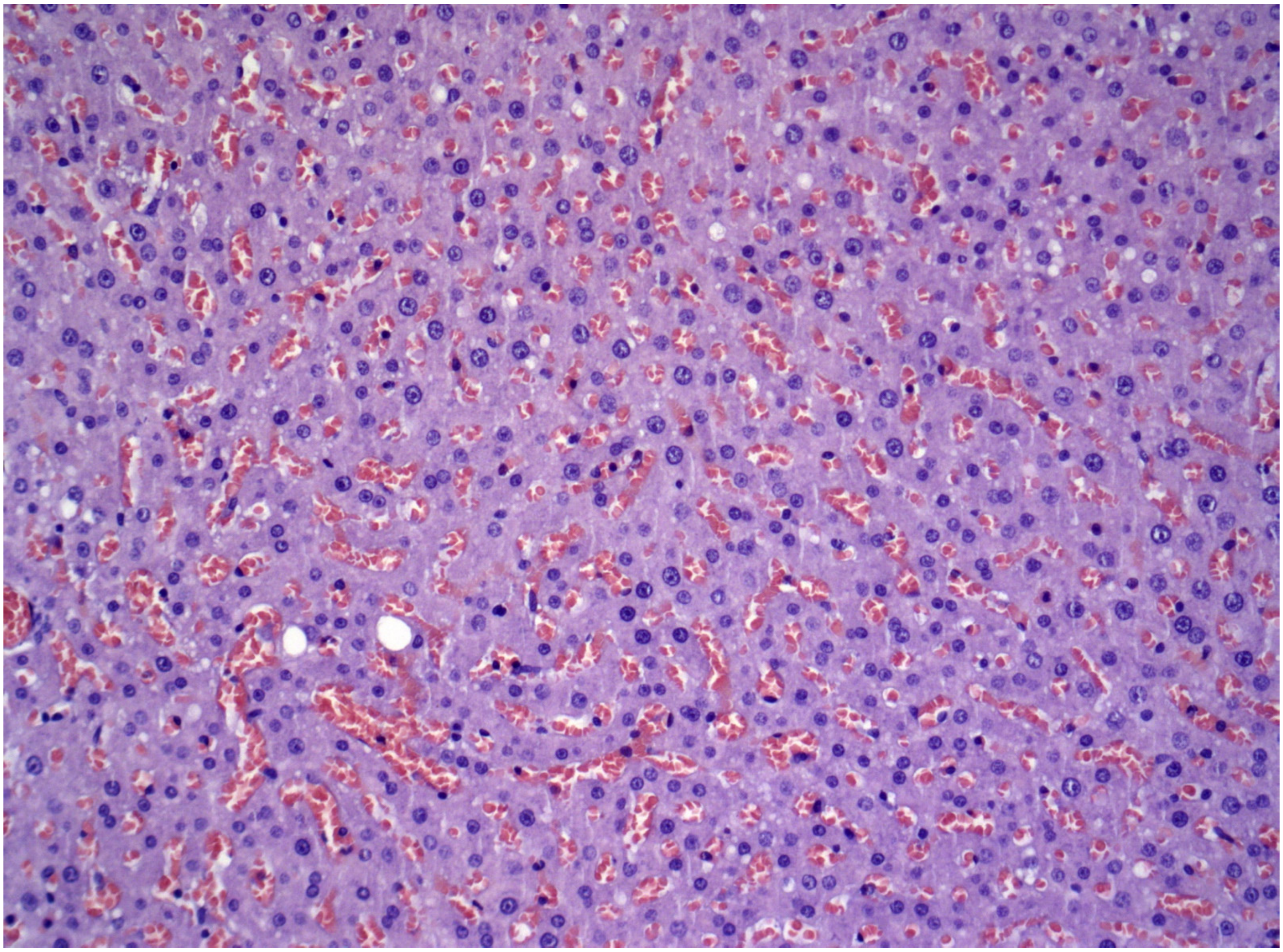

Supplementary Figure 3a

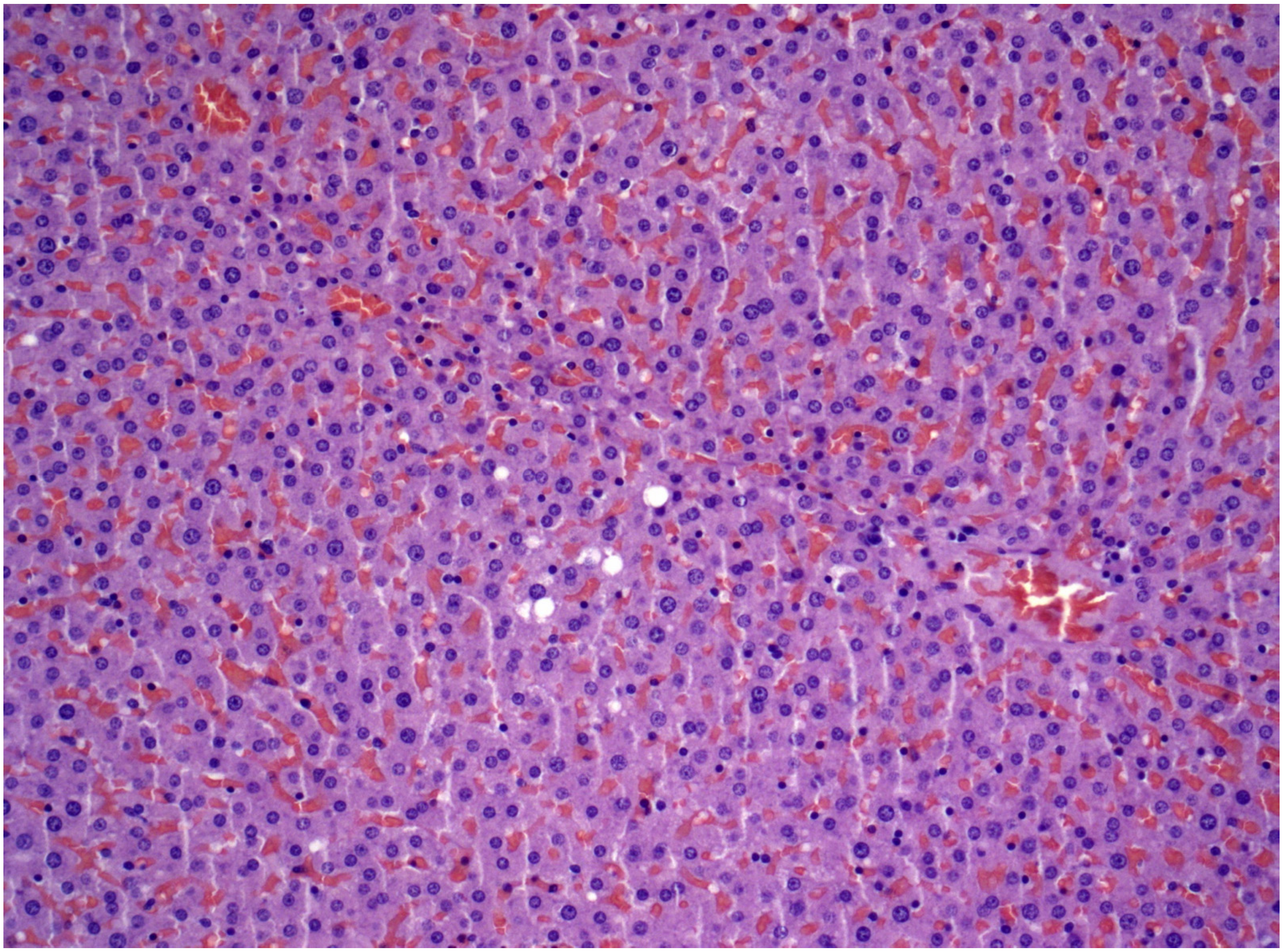

Supplementary Figure 3b

Supplementary Figure 4. Various pathologies observed during histological examination in animals of the control group in the STZ – induced model of type 2 diabetes mellitus that received sodium chloride (diabetes without treatment): **(a, b)** Medium and small lipid droplets with hepatocyte degeneration, **(c)** large and medium lipid droplets with hepatocyte regeneration, **(d)** total autolysis of kidney tissue, **(e)** partial autolysis of the pancreas, kidneys, spleen, and lung, **(f, g)** hepatocyte degeneration. Hematoxylin and eosin staining. Magnitude x400.

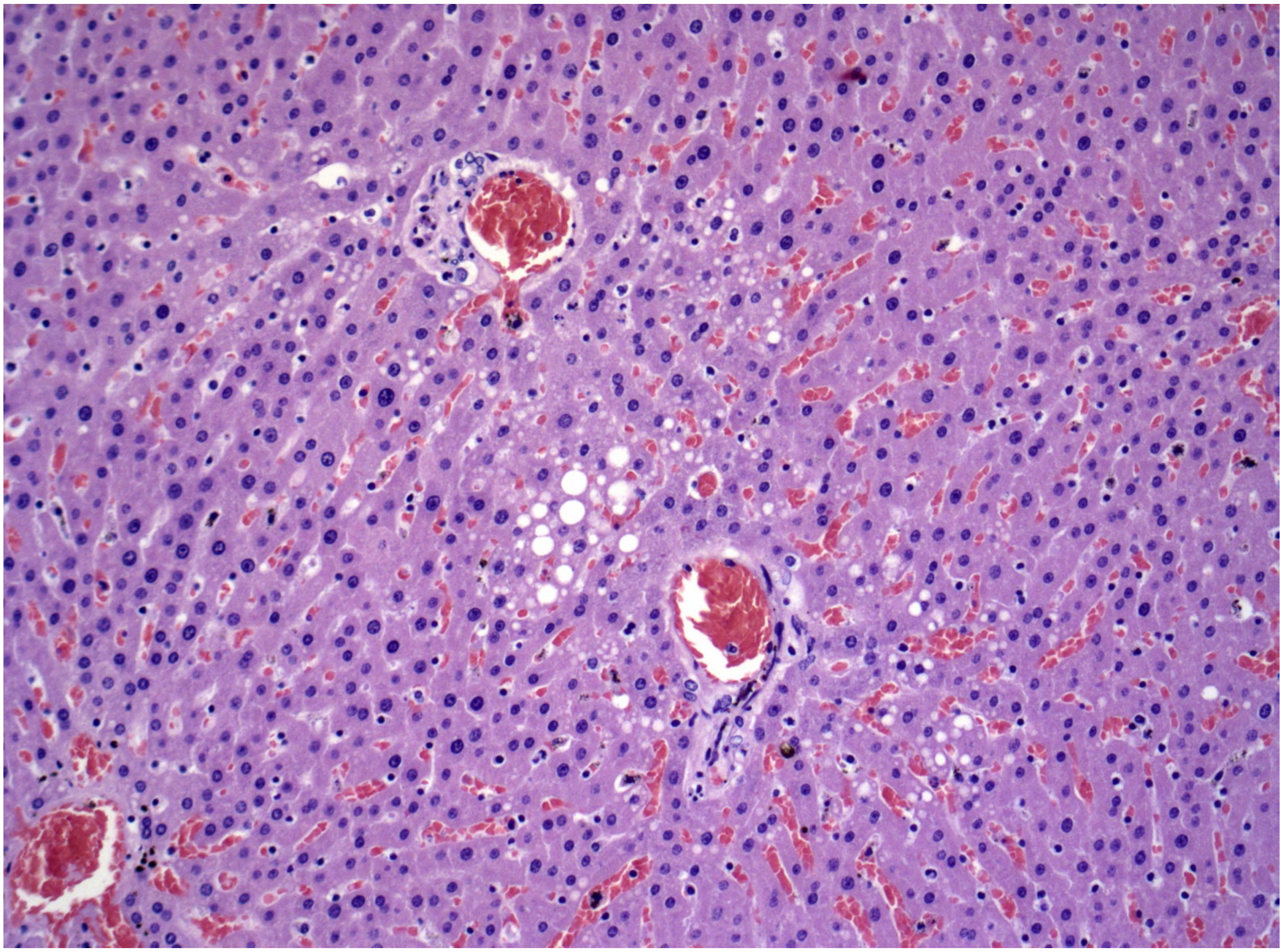

Supplementary Figure 4a

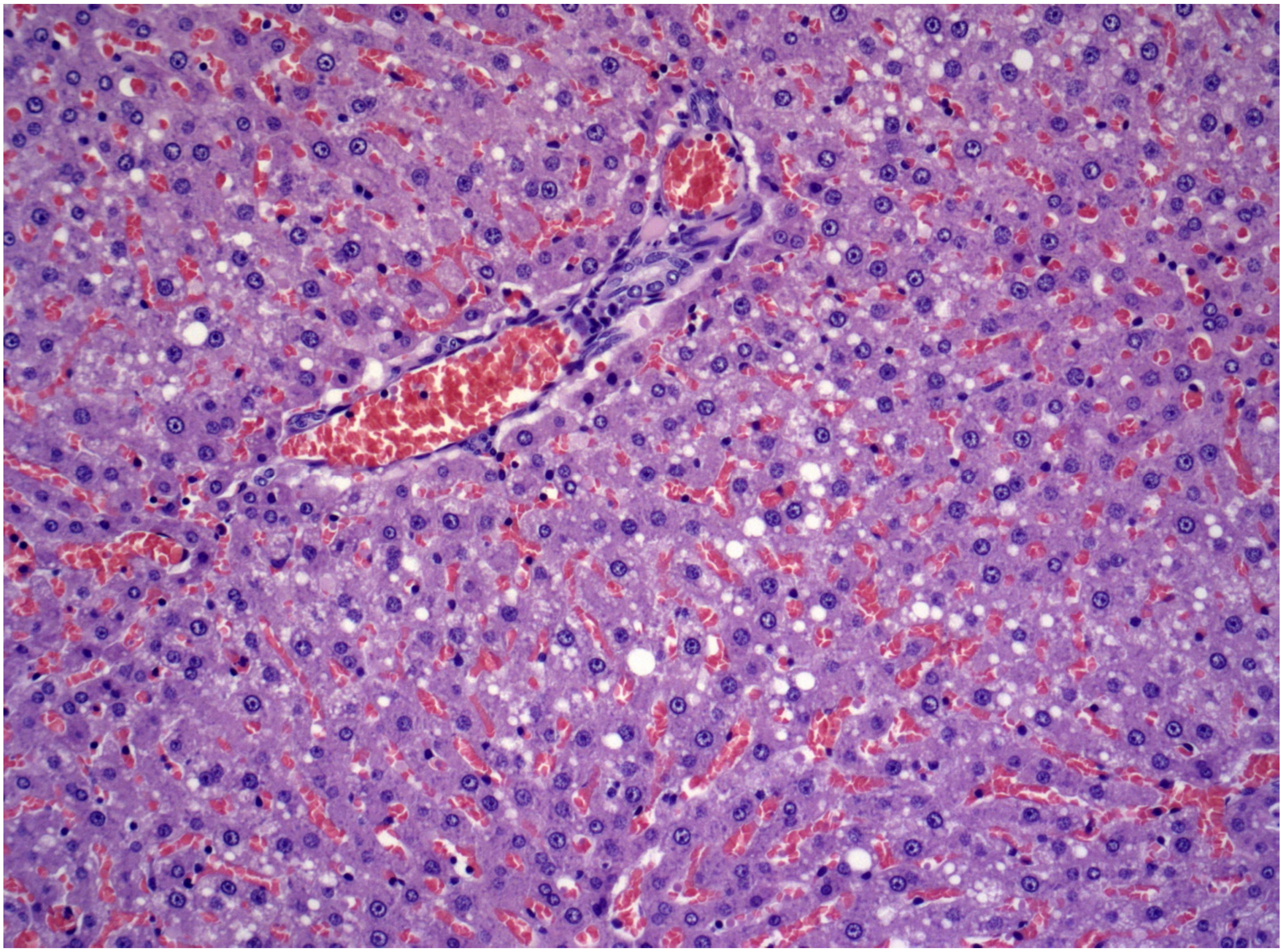

Supplementary Figure 4b

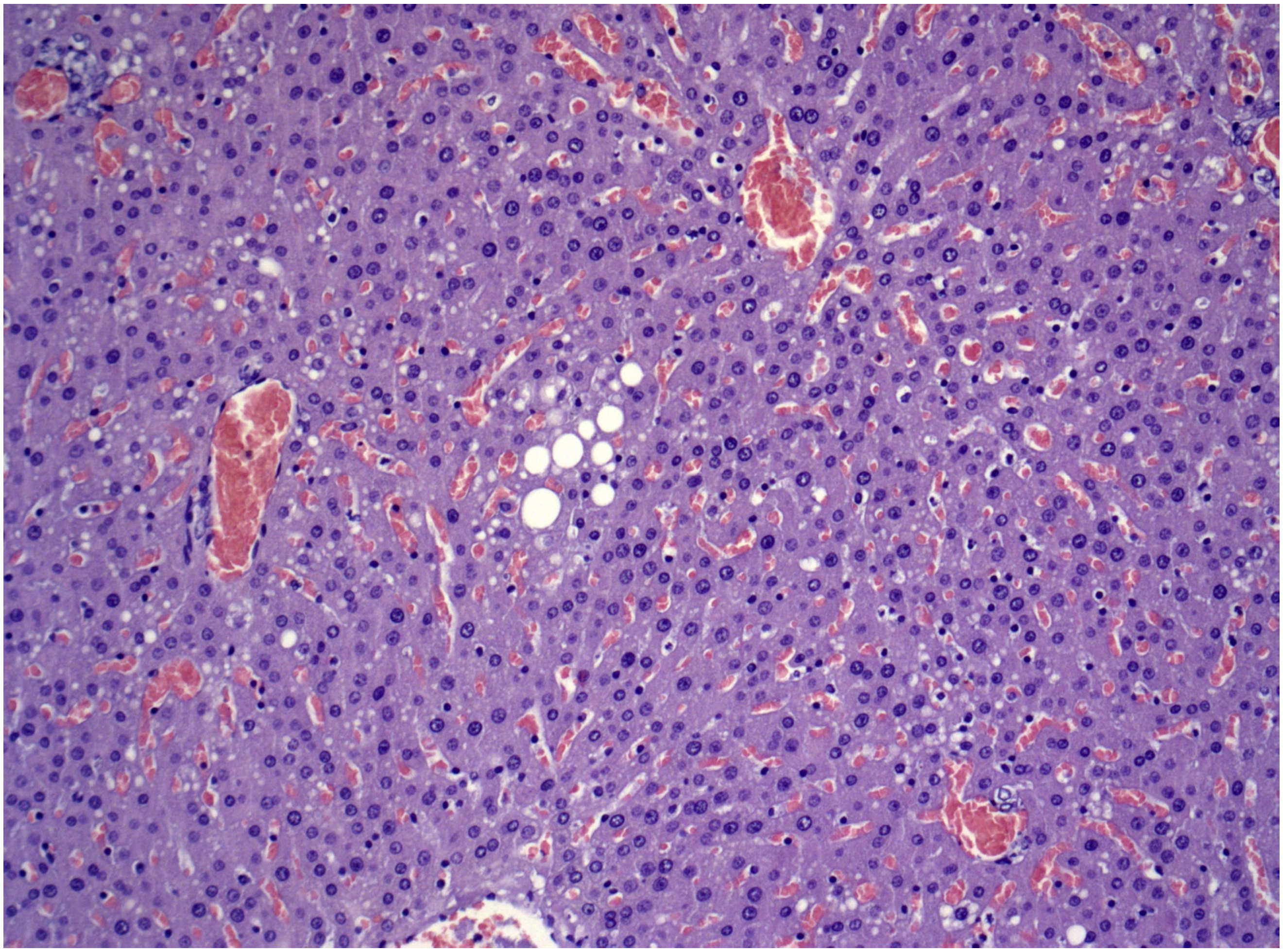

Supplementary Figure 4c

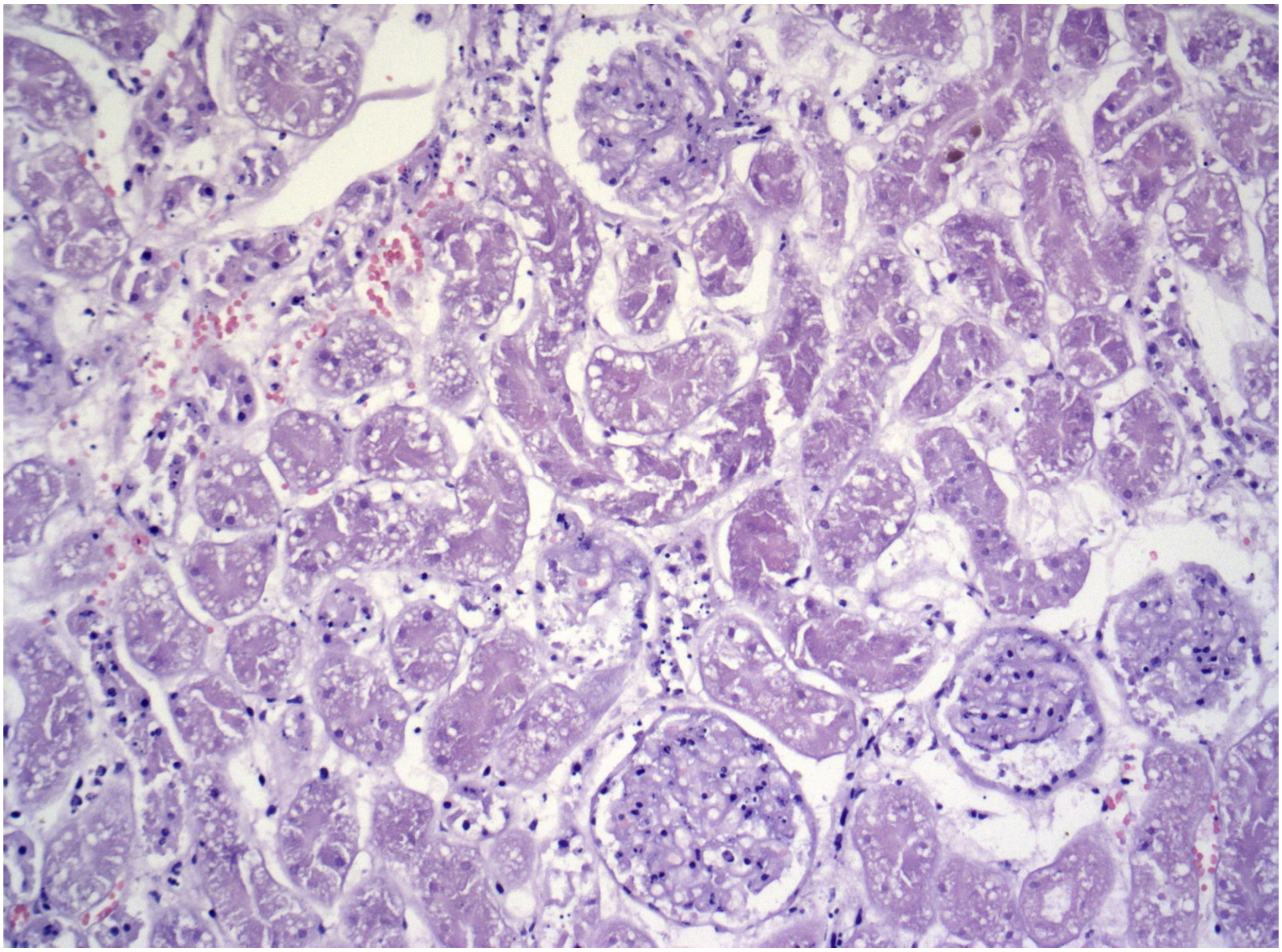

Supplementary Figure 4d

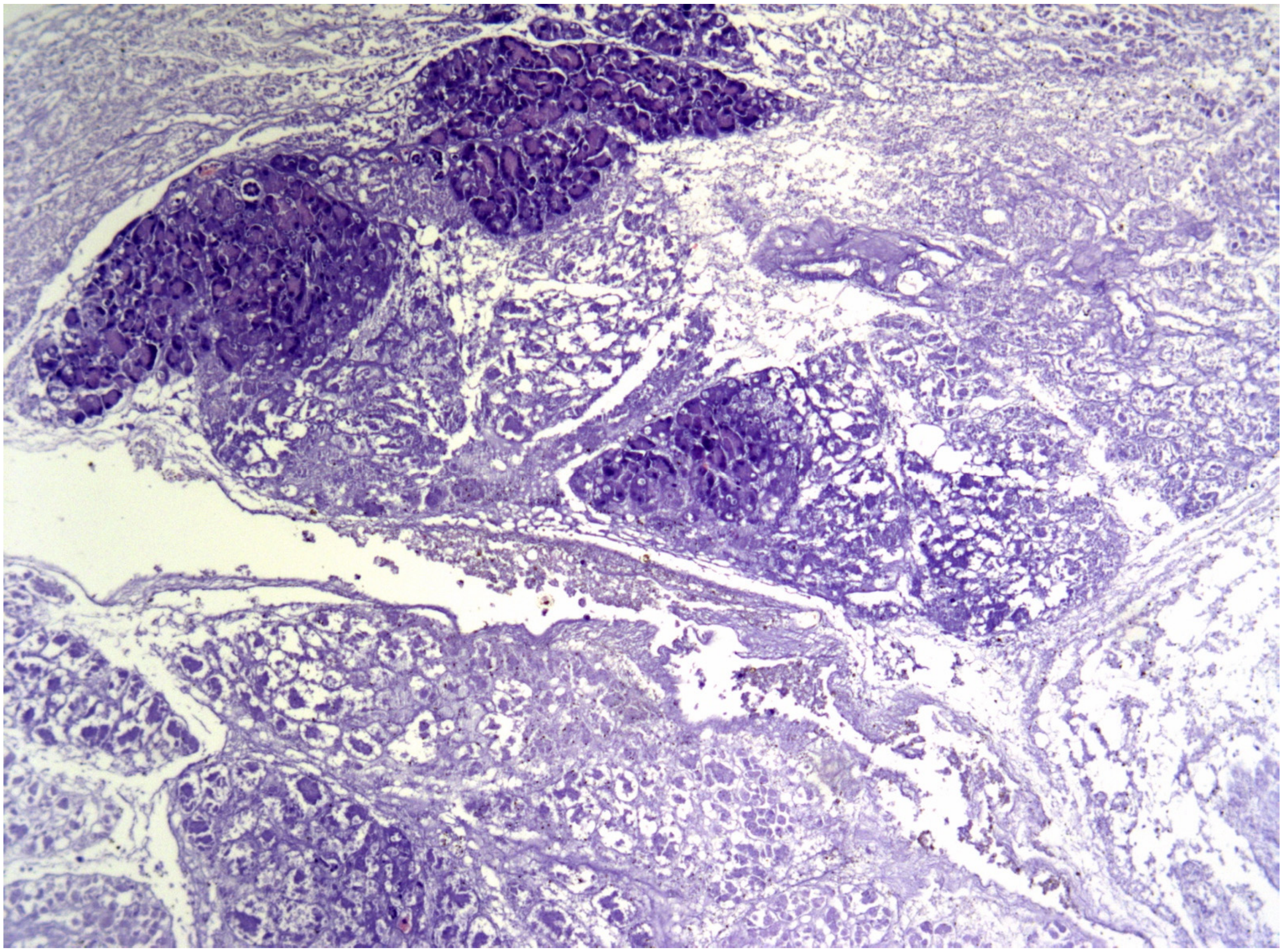

Supplementary Figure 4e

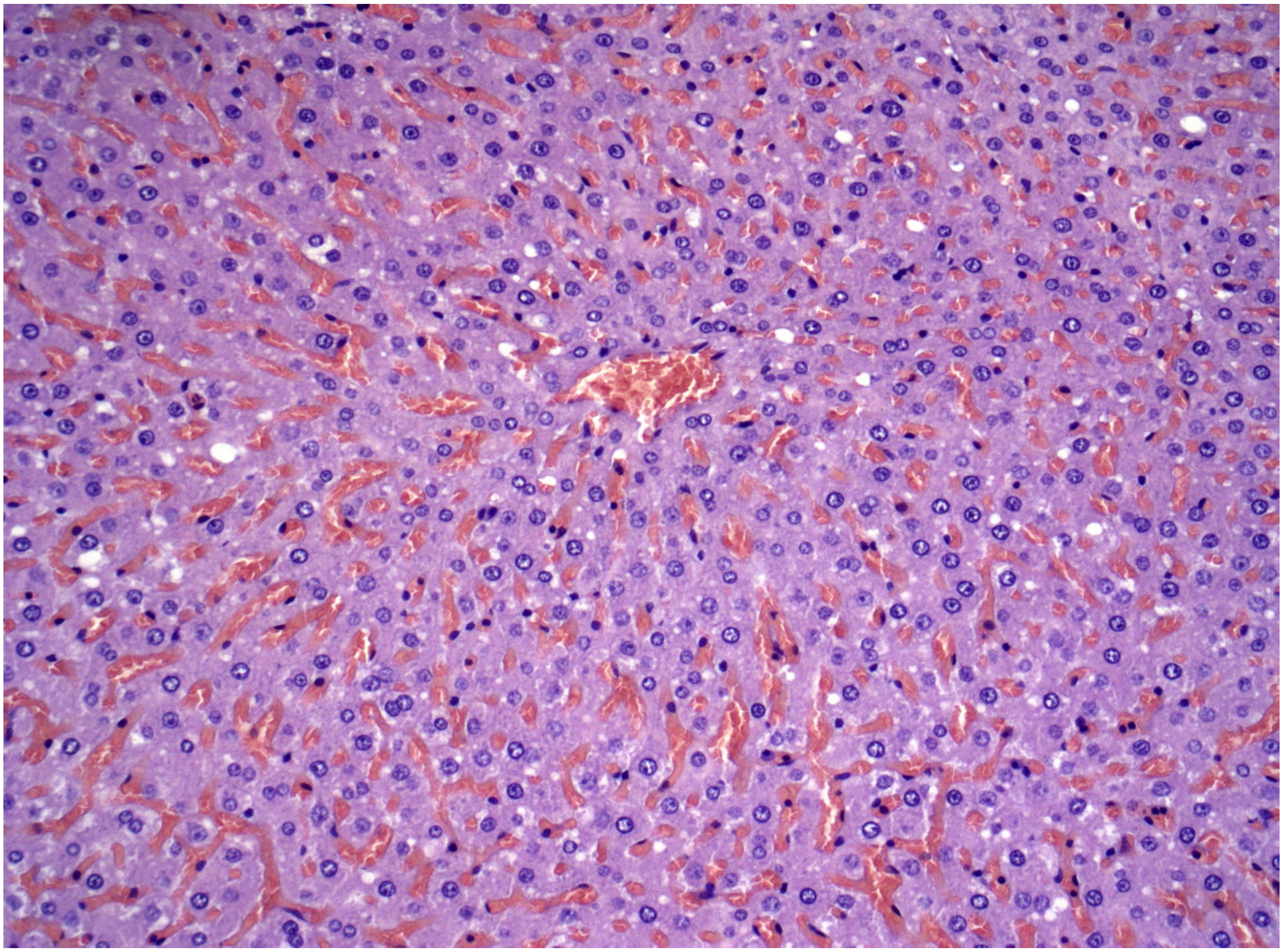

Supplementary Figure 4f

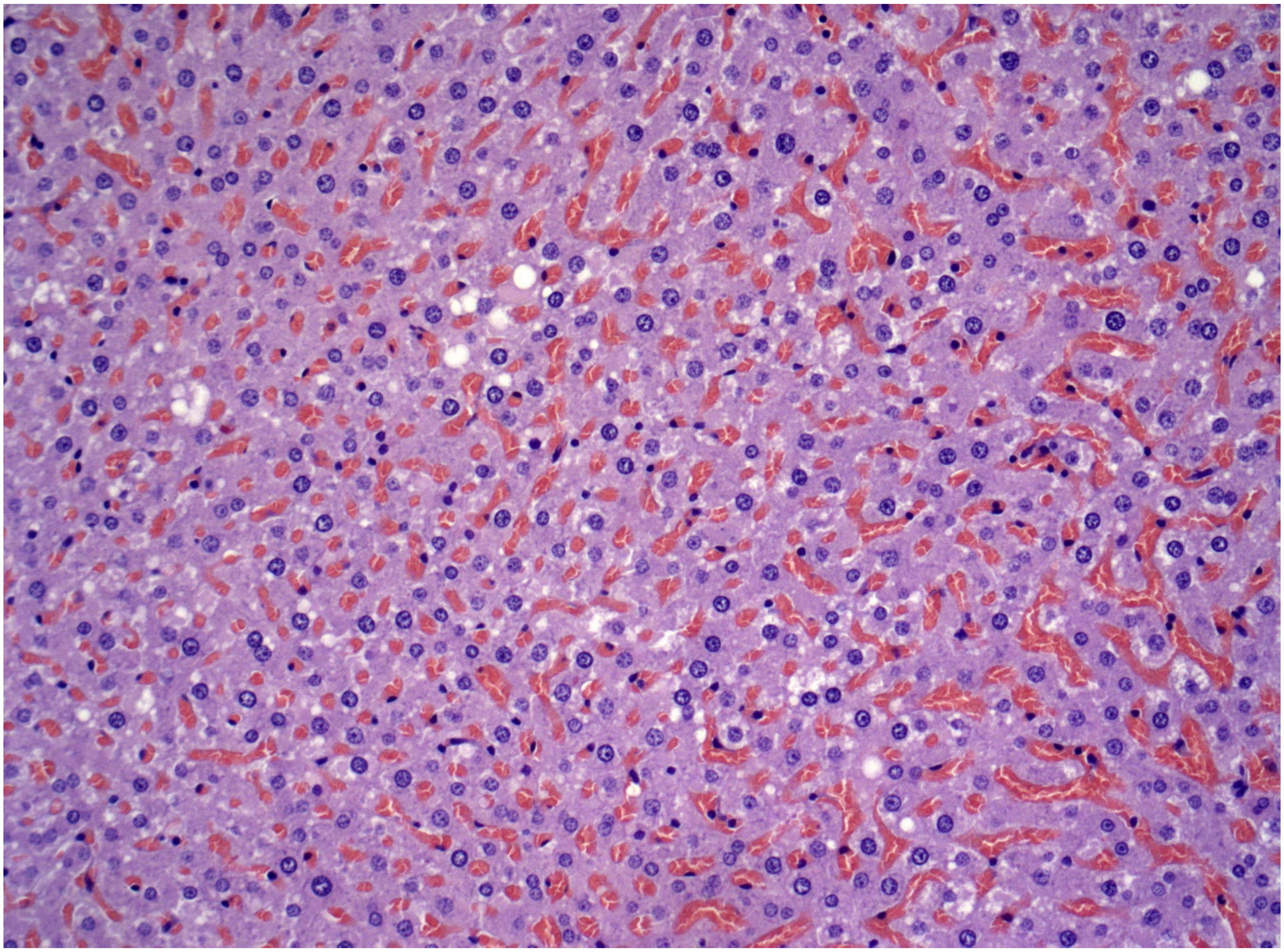

Supplementary Figure 4g
